# Smartwatch Use and Physician Well-Being: A Randomized Clinical Trial

**DOI:** 10.1001/jamanetworkopen.2025.27275

**Published:** 2025-08-18

**Authors:** Liselotte N. Dyrbye, Colin P. West, Angelina R. Wilton, Daniel V. Satele, Arjun P. Athreya

**Affiliations:** 1 Department of Medicine, University of Colorado School of Medicine, Denver; 2 Department of Medicine, Mayo Clinic, Rochester, Minnesota; 3 Department of Quantitative Health Sciences, Mayo Clinic, Rochester, Minnesota; 4 Mayo Clinic Graduate Research Education Program, Mayo Clinic, Rochester, Minnesota; 5 Now with University of Illinois College of Medicine, Chicago; 6 Department of Quantitative Health Sciences, Mayo Clinic, Rochester, Minnesota; 7 Department of Molecular Pharmacology and Experimental Therapeutics, Center for Individualized Medicine, Mayo Clinic, Rochester, Minnesota

## Abstract

**Importance:**

Burnout remains prevalent among physicians and can negatively affect quality, safety, and cost of patient care. Few randomized studies on interventions to address burnout have been conducted to date.

**Objective:**

To determine whether wearing a smartwatch and having access to its physiological data (eg, sleep, step count, and heart rate) improves physician well-being (and if so, which dimensions of well-being).

**Design, Setting, and Participants:**

This randomized clinical trial included physicians at 2 US medical centers (Mayo Clinic and the University of Colorado School of Medicine) who volunteered to wear a provided smartwatch beginning June 7, 2023. The study concluded on June 27, 2024.

**Intervention:**

Six months of wearing a smartwatch.

**Main Outcomes and Measures:**

Burnout, resilience, quality of life, depressive symptoms, stress, and sleepiness were measured using validated scales. Participants completed electronic surveys at baseline and 3, 6, 9, and 12 months. An intention-to-treat analysis was conducted.

**Results:**

This study included 184 physicians (mean [SD] age, 37.5 [9.3] years; 107 females [58.8%]). A total of 83 of 183 physicians (45.4%) were residents or fellows, 103 of 184 (56.0%) lived in Colorado, and 99 of 182 (54.4%) worked in non–primary care settings. Baseline levels of well-being across the measured dimensions were similar for the 2 study arms. At 6 months, 35 of 85 physicians (41.2%) in the intervention arm had burnout compared with 46 of 91 (50.5%) in the control arm (*P* = .21). Mean (SD) resilience scores at 6 months were 31.9 (5.0) and 29.5 (6.2) among physicians in the intervention and control arms, respectively (*P* = .01). In multivariable analysis adjusting for baseline score, demographics, specialty, and work hours, the prevalence of burnout was lower (odds ratio, 0.46 [95% CI, 0.21-0.99]; *P* = .046) and mean resilience score was higher (parameter estimate [scale, 0-40], 1.20 points [95% CI, 0.11-2.28 points]; *P* = .03; Cohen *d* = 0.17) among physicians in the intervention arm vs the control arm after 6 months.

**Conclusions and Relevance:**

In this randomized clinical trial to evaluate the effect of wearing a smartwatch and having access to its physiological data on physician well-being, physicians who wore a smartwatch experienced improvements in burnout and resilience. Future research should explore whether engagement with smartwatch data leads to actual behavior change (eg, adaptive coping or reflective habits) that reduces burnout risk and enhances resilience.

**Trial Registration:**

ClinicalTrials.gov Identifier: NCT05463250

## Key Points

**Question:**

Does wearing a smartwatch and having access to its physiological data (eg, sleep, step count, and heart rate) improve the well-being of physicians?

**Findings:**

In this randomized clinical trial including 184 physicians, burnout was lower and resilience was higher at 6 months among physicians in the intervention arm vs the control arm after adjusting for baseline score, demographics, specialty, and work hours.

**Meaning:**

Wearing a smartwatch and having access to its data may be a worthwhile individual strategy that improves physicians’ well-being.

## Teaser

This randomized clinical trial examines the effects of wearing a smartwatch and having access to its physiological data on physician well-being.

## Introduction

In a 2022 national study of US physicians,^2^ 63% experienced burnout and rates remain higher than other US workers, a finding first documented in 2011.^1^ This persistently high prevalence of burnout among physicians is concerning because it threatens ongoing efforts to improve quality, safety, and cost of care as well as access to care. For example, physicians with burnout are more likely to report recent medical errors, involvement in a medical malpractice suit, behaviors inconsistent with high standards of professionalism, and bias against Black patients.^3,4,5,6^ Additionally, longitudinal studies have reported higher odds of turnover and reduction in clinical hours among physicians with burnout relative to those without burnout, conservatively contributing $4.6 billion in costs to the US health care system annually.^7,8,9,10,11^ On a personal front, physicians with burnout have a higher risk for alcohol use disorder, suicidal ideation, and motor vehicle incidents.^5,6^

In response, the National Academy of Medicine consensus study on clinician burnout called upon leaders in health care organizations and health professions, educational institutions, as well as within the government and industry to prioritize, prevent, and mitigate burnout and foster professional well-being for the overall health of individuals and the nation.^6^ Effective action, however, is hampered by relatively few robust intervention studies having been conducted to guide strategies to reduce burnout and promote well-being among physicians.^12^

According to the World Health Organization, burnout is an occupational phenomenon that results from chronic, high levels of occupational stress.^13^ The job demands-resources model proposes that it is the balance between job demands (eg, workload) and job resources (eg, autonomy), filtered through an individual’s capacity to effectively deal with work stressors, that determines where an individual falls on the spectrum from thriving at work to suffering from a high level of burnout.^6^ Personal choices related to sleep, exercise, and other stress-reducing behaviors have been previously demonstrated to relate to the risk of burnout among physicians.^14,15,16^

Smartwatches are a wearable digital health technology that provide quantitative measures of one’s physiological functioning (eg, heart rate, sleep cycles, and sleep quality) and behaviors (eg, physical activity and sleep length per hour in bed). Some studies suggest that wearing a smartwatch and having access to its data is associated with favorable health behavior changes.^17,18^ Information on physiological functioning collected from smartwatches may encourage physicians to engage in proactive interventions that reduce stress and mitigate their risk of burnout before personal suffering or negative impact to patient care occurs. Having access to personalized physiological data could promote greater self-awareness and self-regulation—key components of well-being. Drawing from cognitive-behavior and self-determination theories, data from smartwatches may help individuals recognize patterns in their behavior, make incremental adjustments aligned with their values or goals, and develop a sense of control or agency, goal-directed behavior, and self-efficacy, all of which have been associated with better well-being in prior literature.^19,20,21^

Despite the widespread use of smartwatches, few studies have explored relationships between having access to data from a wearable device and physician burnout or other dimensions of physician well-being.^22^ Among the limited number of studies conducted with physicians, no association has been found between wearing a wearable health-tracking device and the severity of burnout. These studies, however, included small samples of physicians or residents, collected data over a short period (2-16 weeks), and did not include a control group.^23,24,25,26^ Finally, studies with smartwatches have often suffered from poor adherence (ie, not consistently wearing the smartwatches over extended periods of time) and reduced participant engagement.^22^

We conducted a multisite randomized clinical trial to evaluate whether wearing a smartwatch improves overall well-being among physicians (and if so, which dimensions of well-being; eg, fatigue, stress, overall quality of life, or burnout). We hypothesized that physicians with individualized access to their physiological and physical activity data would engage more frequently in behaviors that mitigate stress, resulting in improved well-being.

## Methods

This randomized clinical trial was approved by the institutional review boards (IRBs) of the University of Colorado School of Medicine in Aurora, Colorado, and the Mayo Clinic in Rochester, Minnesota, and was conducted between June 7, 2023, and June 27, 2024. Participants electronically provided written informed consent. This study was registered at ClinicalTrials.gov (NCT05463250). The trial protocol is presented in Supplement 1. This study followed the Consolidated Standards of Reporting Trials (CONSORT) reporting guideline.

### Study Design, Setting, and Participants

Physicians (residents, fellows, or attending physicians) were eligible to participate in this trial if they met the following criteria: worked at the University of Colorado School of Medicine or at the Mayo Clinic with no anticipated departure within 18 months of enrollment into the study, were employed at least 60% full-time equivalent, and had a smartphone. The study planned to recruit 184 physicians because this would provide 80% power to detect a moderate 0.5-SD to 0.6-SD effect size, a level considered clinically significant.^27^ Participants were recruited through IRB-approved electronic communications, flyers, and announcements, and they electronically provided written informed consent. Interested physicians were requested to complete an electronic screening form, which asked them to provide their name, work location, specialty, and job role (resident or fellow vs attending physician). Offers of study enrollment were sent by email to interested and eligible physicians. Those who completed the consent and baseline survey were considered accrued into the study.

### Randomization, Allocation Concealment, and Follow-Up

Consenting physicians were randomly assigned via a computer-generated algorithm to either the smartwatch immediate intervention arm or the smartwatch delayed intervention arm (control). Randomization was stratified by specialty (primary care [general internal medicine, family medicine, or pediatrics], nonprimary care [internal medicine subspecialty, pediatric subspecialty, dermatology, neurology, physical medicine and rehabilitation, psychiatry, radiation oncology, or emergency medicine], and surgery [anesthesia, obstetrics and gynecology, or surgery specialties]), work site (Colorado or Minnesota), and physician category (resident or fellow vs attending physician) using permuted blocks. Participants were asked to complete electronic surveys at baseline and at 3, 6, 9, and 12 months.

### Study Arms

After enrollment and completion of the baseline survey, participants randomized to the immediate intervention arm received a smartwatch (Venu 2 Plus; Garmin) and an electronic smartwatch manual with step-by-step instructions for setting up linkages with the smartwatch app (Garmin Connect) and a cloud-based data aggregation service (Fitabase; SmallSteps LLC). They also received IRB-approved newsletters via email every other month throughout the study. These newsletters featured aggregated statistics of data collected from the smartwatches, reminders to sync their smartwatch data, and information about smartwatch features. Participants randomized to the delayed intervention arm received no intervention for the first 6 months of the study. After completion of the 6-month survey, they received a smartwatch (Venu 3S; Garmin [the model given to the immediate intervention arm was no longer available]) and an electronic smartwatch manual, as well as the IRB-approved newsletters via email every other month. Both study smartwatches tracked heart rate, activity, respiratory rate, stress levels, and sleep patterns and were compatible with the 2 most prominent smartphone ecosystems (iOS and Android).

### Adherence, Technology Support, and Participant Engagement Strategies

Using the cloud-based data aggregation service, the study team monitored adherence, defined by wear time (ie, percentage of time the smartwatch wrist sensor detected a pulse over 24 hours). If no data were seen for 14 days, study team members reached out to remind participants to synchronize their data (ie, to send smartwatch data from their smartphone to the cloud-based data aggregation service) and asked whether there were any problems with the smartwatch. Technical support was available to study participants via email. Three watches were replaced due to malfunctioning or accidental damage. Toward the final months of the study (March 1-May 31, 2024), participants whose wear time was at least 70% received a $25 e-gift card.

### Outcomes and Measures

The surveys included questions about self-reported demographics (age, gender identity, race and ethnicity, and relationship status) and professional characteristics, with validated instruments to measure the primary outcome (burnout) and secondary outcomes (resilience, quality of life, depressive symptoms, stress, and sleepiness). Race was included as a survey item to assess potential differential effects of the burnout intervention across racial groups, given that prior studies have identified differences in the prevalence of burnout by demographics, including race and ethnicity.^42^ Participants selected all race and ethnicity categories that applied, including American Indian or Alaska Native, Asian, Black or African American, Hispanic or Latino, Native Hawaiian or Other Pacific Islander, White, or other race (self-selected category). Individuals who indicated more than 1 race were categorized as multiracial. The Mayo Clinic Survey Research Center administered the electronic surveys at baseline and at 3, 6, 9, and 12 months. Participants received $25 for each survey completed.

Burnout was measured with the emotional exhaustion (range, 0-54) and depersonalization (range, 0-30) subscales of the Maslach Burnout Inventory (MBI; higher scores indicate greater burnout symptoms),^28^ applied under a license with Mind Garden Inc. Additionally, scores were dichotomized, with 27 or higher on the MBI emotional exhaustion subscale and 10 or higher on the MBI depersonalization subscale indicating a high score. Overall burnout was defined as having a high score on either the MBI emotional exhaustion subscale or the depersonalization subscale, consistent with prior studies.^1,2,3,4,5^

Resilience was measured using the 10-item Connor-Davidson Resilience Scale (range, 0-40), with higher scores indicating greater resilience.^29,30^ Quality of life was measured using a single-item linear analog scale (range, 0-10), with higher scores indicating better quality of life.^31,32,33,34^ Depressive symptoms were measured using the Patient-Reported Outcomes Measurement Information System 4a short form.^35,36^ Stress was measured using the 10-item Perceived Stress Scale (range, 0-40), with higher scores indicating greater stress.^37^ Sleepiness was measured using the Epworth Sleepiness Scale (range, 0-24), with higher scores suggesting more sleepiness.^38^ Each of these metrics has been validated across a wide range of medical conditions and populations, including physicians.

### Statistical Analysis

We calculated standard univariate statistics to characterize the sample. We made unadjusted comparisons between groups using χ^2^ or Kruskal-Wallis tests, as appropriate. Within groups, changes in each measure from baseline to 6 months were compared using paired *t* tests or McNemar tests, as appropriate. Multivariable models were analyzed for each outcome (burnout, quality of life, depressive symptoms, stress, sleepiness, and resilience) comparing study arms adjusted for baseline outcome value, site, specialty, physician category, self-reported hours worked per week, and demographics at 6 months. All tests were 2-sided, and statistical significance was set at *P* < .05. We used SAS, version 9.4 (SAS Institute), and R, version 4.4.2 (R Project for Statistical Computing), for all analyses.

## Results

### Sample Characteristics and Baseline Measures

This study included 184 physicians (mean [SD] age, 37.5 [9.3] years) (Figure). A total of 107 physicians (58.8%) identified as female and 75 (41.2%) identified as male (gender identity was missing for 2 physicians [0.01%]) (Table 1). Race was available for 181 physicians; 1 (0.6%) identified as American Indian or Alaska Native, 19 (10.5%) as Asian, 4 (2.2%) as Black or African American, 151 (83.4%) as White, 2 (1.1%) as other race, and 4 (2.2%) as multiracial. Ethnicity was available for 183 physicians; 21 (11.5%) identified as Hispanic or Latino and 162 (88.5%) identified as non-Hispanic or non-Latino. A total of 83 of 183 physicians (45.4%) were residents or fellows, 103 of 184 (56.0%) lived in Colorado, and 99 of 182 (54.4%) worked in non–primary care settings. There were no statistically significant differences in baseline demographics or professional characteristics between participants randomized to the intervention or the control. Baseline levels of burnout, quality of life, depressive symptoms, stress, sleepiness, and resilience also did not statistically differ among participants in the 2 arms.

**Figure.**
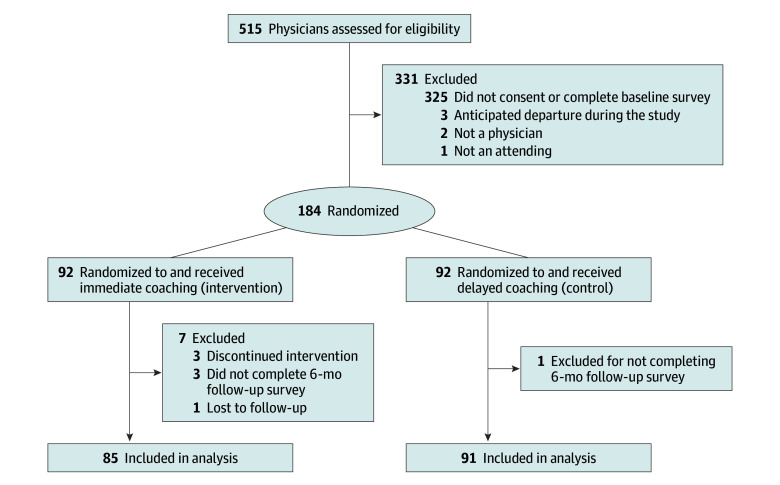
Study Flow Diagram

**Table 1.** Baseline Characteristics of Participants^a^

Characteristic	Immediate intervention arm (n = 92)	Delayed intervention arm (n = 92)
Role		
Attending physician	51/91 (56.0)	49/92 (53.3)
Resident or fellow	40/91 (44.0)	43/92 (46.7)
Site		
Colorado	52/92 (56.5)	51/92 (55.4)
Minnesota	40/92 (43.5)	41/92 (44.6)
Specialty^b^		
Non–primary care	48/91 (52.7)	51/91 (56.0)
Primary care	22/91 (24.2)	23/91 (25.3)
Surgery	21/91 (23.1)	17/91 (18.7)
Postgraduate year of training (residents or fellows only)		
1	6/40 (15.0)	6/43 (14.0)
2	16/40 (40.0)	21/43 (48.8)
3	6/40 (15.0)	6/43 (14.0)
≥4	12/40 (30.0)	10/43 (23.3)
Years in practice (attending physicians only)		
Mean (SD)	10.9 (8.3)	10.3 (8.8)
Median (range)	9 (1.0-42.0)	7 (1.0-33.0)
Percentage FTE (attending physicians only)		
50-74	1/51 (2.0)	0
75-99	10/51 (19.6)	9/49 (18.4)
100	40/51 (78.4)	40/49 (81.6)
Percentage FTE dedicated to direct patient care activities (attending physicians only)		
<10	1/51 (2.0)	0
10-24	3/51 (5.9)	2/48 (4.2)
25-49	6/51 (11.8)	6/48 (12.5)
50-74	10/51 (19.6)	12/48 (25.0)
75-99	25/51 (49.0)	22/48 (45.8)
100	6/51 (11.8)	6/48 (12.5)
Work hours per week, mean (SD)	59.6 (11.8)	56.2 (12.5)
Nights on call, mean (SD)	0.9 (1.4)	0.9 (1.0)
Age, mean (SD), y	37.6 (9.0)	37.4 (9.6)
Relationship status		
Single	12/92 (13.0)	7/92 (7.6)
Married	67/92 (72.8)	69/92 (75.0)
Partnered	12/92 (13.0)	16/92 (17.4)
Missing	1/92 (1.1)	0
Race^c^		
American Indian or Alaska Native	0	1/92 (1.1)
Asian	10/89 (11.2)	9/92 (9.8)
Black or African American	3/89 (3.4)	1/92 (1.1)
White	73/89 (82.0)	78/92 (84.8)
Other^d^	1/89 (1.1)	1/92 (1.1)
Multiracial	2/89 (2.2)	2/92 (2.2)
Ethnicity		
Hispanic or Latino	13/91 (14.3)	8/92 (8.7)
Non-Hispanic or non-Latino	78/91 (85.7)	84/92 (91.3)
Gender identity		
Male	35/91 (38.5)	40/91 (44.0)
Female	56/91 (61.5)	51/91 (56.0)
Emotional exhaustion, mean (SD)^e^	23.1 (12.4)	23.7 (10.2)
High	35/90 (38.9)	34/91 (37.4)
Not high	55/90 (61.1)	57/91 (62.6)
Depersonalization, mean (SD)^f^	8.1 (6.9)	8.4 (6.4)
High	32/90 (35.6)	31/91 (34.1)
Not high	58/90 (64.4)	60/91 (65.9)
Overall burnout^g^		
Yes	45/90 (50.0)	39/91 (42.9)
No	45/90 (50.0)	52/91 (57.1)
Resilience, mean (SD)^h^	30.7 (5.3)	29.2 (5.6)
Quality of life, mean (SD)^i^	6.9 (1.6)	6.8 (1.6)
Depressive symptoms, mean (SD)^j^	6.1 (2.9)	6.5 (2.9)
Stress, mean (SD)^k^	15.6 (5.9)	16.3 (5.7)
Sleepiness, mean (SD)^l^	7.2 (4.5)	6.4 (4.4)

Abbreviation: FTE, full-time equivalent.

^a^ Unless indicated otherwise, values are presented as No./total No. (%) of physicians.

^b^ Primary care includes general internal medicine, family medicine, and pediatrics. Nonprimary care includes internal medicine subspecialty, pediatric subspecialty, dermatology, neurology, physical medicine and rehabilitation, psychiatry, radiation oncology, and emergency medicine. Surgical specialties include anesthesia, obstetrics and gynecology, and surgery specialties.

^c^ Participants could choose more than 1 category.

^d^ Self-selected without further subcategorization.

^e^ Maslach Burnout Inventory subscale (score range 0-54, with higher scores indicating greater burnout symptoms; high emotional exhaustion was defined as a score ≥27).

^f^ Maslach Burnout Inventory subscale (score range 0-30, with higher scores indicating greater burnout symptoms; high depersonalization was defined as a score ≥10).

^g^ Positive for symptoms of overall burnout if the physician had high emotional exhaustion, high depersonalization, or both.

^h^ Connor-Davidson Resilience Scale (score range 0-40, with higher scores indicating greater resilience).

^i^ Single-item linear analog scale (score range 0-10, with higher scores indicating better quality of life).

^j^ Patient-Reported Outcomes Measurement Information System 4a short form (score range 4-20, with higher scores indicating greater depressive symptoms).

^k^ Perceived Stress Scale (score range 0-40, with higher scores indicating more stress).

^l^ Epworth Sleepiness Scale (score range 0-24, with higher scores indicating more sleepiness).

Among the 92 participants in the intervention arm, 86 (93.5%), 85 (92.4%), and 85 (92.4%) responded to the 3-month, 6-month, and 12-month surveys, respectively. Among the 92 participants in the control arm, 88 (95.7%), 91 (98.9%), and 91 (98.9%) responded to the 3-month, 6-month, and 12-month surveys, respectively. Physicians in the immediate smartwatch intervention arm had a mean (SD) daily wear time proportion of 76.1% (33.3%) from August 1, 2023, through December 31, 2023, a time point corresponding to the 6-month survey (eFigure in Supplement 2). Over the remaining study months leading to the 12-month survey, the mean (SD) daily wear time proportion was 73.6% (36.4%) and 73.8% (37.2%) for the physicians in the immediate intervention and delayed intervention arms, respectively.

### Randomized Arms

Burnout, resilience, quality of life, depression, stress, and sleepiness scores at 3 and 6 months among participants in the 2 arms of the trial are presented in eTable 1 in Supplement 2. At 6 months, overall burnout was lower among physicians in the immediate intervention arm vs the delayed intervention arm, but the difference was not statistically significant (35 of 85 [41.2%] vs 46 of 91 [50.5%]; *P* = .21). On multivariable analysis, physicians in the immediate intervention arm had a 54.0% reduction in the odds of overall burnout (odds ratio, 0.46 [95% CI, 0.21-0.99]; *P* = .046) (Table 2). Mean emotional exhaustion and depersonalization scores favored the intervention but did not differ statistically between the study arms after adjusting for baseline outcome value, site, specialty, hours worked per week, role, and demographics (eTable 2 in Supplement 2). Mean (SD) resilience score was higher among those in the immediate intervention arm vs the delayed intervention arm (31.9 [5.0] vs 29.5 [6.2]; *P* = .01). On multivariate analysis, mean resilience score was higher among those in the immediate intervention arm vs the delayed intervention arm at 6 months (parameter estimate [0-40 scale], 1.20 points [95% CI, 0.11-2.28 points]; *P* = .03; Cohen *d* = 0.17) (Table 2). No statistically significant differences were seen in quality of life, depressive symptoms, stress, and sleepiness scores (eTable 3 in Supplement 2).

**Table 2.** Multivariable Models for Burnout and Resilience at 3 and 6 Months

Variable	Month 3			Month 6		
	Effect size (95% CI)^a^	*P* value		OR or PE (95% CI)	*P* value	
		Class	Overall		Class	Overall
**Burnout, odds ratios**						
Baseline burnout	31.58 (12.13-82.21)	NA	<.001	11.52 (5.17-25.65)	NA	<.001
Arm: Intervention (vs control)	1.14 (0.47-2.76)	NA	.77	0.46 (0.21-0.99)	NA	.046
Role: Attending physician (vs resident or fellow)	0.80 (0.22-2.95)	NA	.74	0.52 (0.18-1.54)	NA	.24
Site: Colorado (vs Minnesota)	0.89 (0.35-2.25)	NA	.80	0.56 (0.25-1.27)	NA	.17
Specialty (vs primary care)						
Non–primary care	0.90 (0.31-2.62)	.85	.81	0.73 (0.29-1.88)	.52	.73
Surgery	1.37 (0.33-5.69)	.67		0.64 (0.20-2.09)	.46	
Work hours per week (for each additional hour)	1.03 (0.99-1.07)	NA	.16	1.01 (0.97-1.04)	NA	.69
Age (for each additional year)	1.04 (0.98-1.12)	NA	.19	1.00 (0.95-1.06)	NA	.93
Gender identity: Female (vs male)	2.54 (1.03-6.28)	NA	.04	1.29 (0.60-2.77)	NA	.52
Relationship status (vs single)						
Married	0.97 (0.22-4.23)	.97	.14	2.31 (0.61-8.79)	.22	.14
Partnered	4.08 (0.67-24.69)	.13		4.67 (1.02-21.41)	.05	
Race: White (compared with all other races)	0.71 (0.23-2.22)	NA	.55	0.77 (0.29-2.02)	NA	.60
Ethnicity: Non-Hispanic or non-Latino (compared with Hispanic or Latino)	2.55 (0.60-10.79)	NA	.20	0.71 (0.21-2.41)	NA	.58
**Resilience, parameter estimates**						
Baseline resilience	0.67 (0.55-0.79)	NA	<.001	0.80 (0.70-0.91)	NA	<.001
Arm: Intervention (vs control)	2.67 (1.44-3.91)	NA	<.001	1.20 (0.11-2.28)	NA	.03
Role: Attending physician (vs resident or fellow)	−0.08 (−1.83 to 1.68)	NA	.93	−0.08 (−1.64 to 1.49)	NA	.92
Site: Colorado (vs Minnesota)	−0.23 (−1.49 to 1.04)	NA	.73	−0.47 (−1.58 to 0.64)	NA	.40
Specialty (vs primary care)						
Nonprimary care	0.99 (−0.52 to 2.50)	.20	.27	0.12 (−1.24 to 1.48)	.86	.31
Surgery	1.49 (−0.42 to 3.39)	.13		1.14 (−0.54 to 2.82)	.18	
Work hours per week (for each additional hour)	−0.01 (−0.06 to 0.05)	NA	.81	−0.03 (−0.08 to 0.01)	NA	.17
Age (for each additional year)	0.04 (−0.05 to 0.13)	NA	.38	−0.03 (−0.11 to 0.05)	NA	.50
Gender identity: Female (vs male)	−0.23 (−1.49 to 1.02)	NA	.71	−0.76 (−1.85 to 0.34)	NA	.18
Relationship status (vs single)						
Married	0.84 (−1.38 to 3.05)	.46	.76	0.15 (−1.77 to 2.07)	.88	.10
Partnered	0.71 (−1.84 to 3.26)	.58		−1.59 (−3.81 to 0.63)	.16	
Race: White (compared with all other races)	0.50 (−1.12 to 2.11)	NA	.55	0.33 (−1.08 to 1.73)	NA	.65
Ethnicity: Non-Hispanic or non-Latino (compared with Hispanic or Latino)	−0.66 (−2.72 to 1.39)	NA	.52	0.65 (−1.16 to 2.46)	NA	.48

Abbreviations: NA, not applicable; OR, odds ratio; PE, parameter estimate.

^a^ ORs are presented for all burnout variables, whereas PEs are given for all resilience variables.

From 6 to 12 months, the numeric improvements in overall burnout, emotional exhaustion, and depersonalization continued among participants in the immediate intervention arm, but the gains were not statistically significant (Table 3). Resilience, quality of life, depressive symptoms, stress, and sleepiness also improved during this time, with mean (SD) quality of life score improving from 6 to 12 months (7.1 [1.9] vs 7.5 [1.8]; *P* = .03; Cohen *d* = 0.23) (Table 3). eTable 4 in Supplement 2 presents the between-group comparisons at 9 and 12 months.

**Table 3.** Paired Analysis: Immediate Intervention Cohort at 0 to 6 Months and 6 to 12 Months^a^

Variable	Month 6 (n = 83)	*P* value for months 0-6	Month 12 (n = 83)	*P* value for months 6-12
Burnout				
Emotional exhaustion, mean (SD)^b^	20.5 (11.5)	.02	18.9 (12.1)	.07
High	27 (32.5)	.62	19 (22.9)	.06
Not high	56 (67.5)		64 (77.1)	
Depersonalization, mean (SD)^c^	6.5 (5.9)	.01	5.7 (5.9)	.06
High	22 (26.5)	.20	18 (21.7)	.25
Not high	61 (73.5)		65 (78.3)	
Overall burnout^d^				
Yes	33 (39.8)	.18	26 (31.3)	.09
No	50 (60.2)		57 (68.7)	
Resilience, mean (SD)^e^	32.2 (4.9)	.01	32.8 (5.0)	.13
Quality of life, mean (SD)^f^	7.1 (1.9)	.64	7.5 (1.8)	.03
Depressive symptoms, mean (SD)^g^	5.7 (2.5)	.51	5.5 (2.8)	.48
Stress, mean (SD)^h^	14.3 (6.4)	.11	13.4 (6.7)	.14
Sleepiness, mean (SD)^i^	6.3 (4.5)	.03	5.8 (4.7)	.12

^a^ Unless indicated otherwise, values are presented as No. (%) of physicians.

^b^ Maslach Burnout Inventory subscale (score range 0-54, with higher scores indicating greater burnout symptoms; high emotional exhaustion was defined as a score ≥27).

^c^ Maslach Burnout Inventory subscale (score range 0-30, with higher scores indicating greater burnout symptoms; high depersonalization was defined as a score ≥10).

^d^ Positive for symptoms of overall burnout if the physician had high emotional exhaustion, high depersonalization, or both.

^e^ Connor-Davidson Resilience Scale (score range 0-40, with higher scores indicating greater resilience).

^f^ Single-item linear analog scale (score range 0-10, with higher scores indicating better quality of life).

^g^ PROMIS 4a short form (score range 4-20, with higher scores indicating greater depressive symptoms).

^h^ Perceived Stress Scale (score range 0-40, with higher scores indicating more stress).

^i^ Epworth Sleepiness Scale (score range 0-24, with higher scores indicating more sleepiness).

From 6 to 12 months, improvements in overall burnout, emotional exhaustion, depersonalization, resilience, and quality of life occurred among participants in the delayed intervention arm, corresponding to when they were wearing the smartwatches (eTable 5 in Supplement 2).

## Discussion

In this randomized clinical trial, physicians who wore smartwatches had lower burnout and higher mean resilience scores at 6 months. Based on previously observed associations, the 54.0% reduction in the odds of overall burnout at 6 months observed in this study could lead to meaningfully lower rates of self-reported medical errors, malpractice litigation, and turnover and lost productivity, along with reduced associated costs to health systems and society.^5,6,11^ The improvement in resilience score exceeded that observed in previous intervention studies.^12,39,40,41^ However, no statistically significant difference was seen in quality of life, depressive symptoms, stress, or sleepiness between the trial arms at 6 months, suggesting wearing a smartwatch and having access to its collected data does not lead to global improvements in well-being. Rather, wearing a smartwatch shows promise as an individual strategy to mitigate burnout and improve resilience, and it should be coupled with other individual and organizational efforts to address well-being more broadly.

The mechanism underlying improvements in burnout and resilience, despite the absence of measurable differences in stress and sleepiness scores, cannot be determined from this study and warrants further investigation. In particular, future research should explore whether engagement with smartwatch data leads to actual behavior change (eg, adaptive coping or reflective habits) that reduce burnout risk and enhanced resilience, or whether the observed benefits stem from increased awareness of one’s physiological state combined with participation in an institutionally sponsored research initiative.

The gains in well-being seen during the first 6 months of this study persisted among the intervention participants during the subsequent 6 months of the study, with mean quality of life score statistically improving from month 6 to month 12 among participants in the immediate intervention. Although they are preliminary, these findings suggest that wearing a smartwatch over longer periods of time could further facilitate behaviors that improve overall quality of life. Additionally, in the delayed intervention cohort, burnout and resilience scores improved from month 6 to month 12, when physicians were wearing the smartwatch, yielding statistically similar outcomes between the 2 arms at 9 and 12 months.

In this study, there was a high retention rate, and the proportion of smartwatch wear time was high in both arms, averaging more than 70.0% for both the immediate intervention participants who wore the smartwatch for 12 months and the delayed intervention participants who wore the watch for 6 months. These retention rates and proportions of wear time were higher than those reported in prior intervention studies.^22^ Although the reasons for these differences are unknown, the decentralized enrollment, smartwatch manual with step-by-step instructions for setting up linkages with the smartwatch app and a cloud-based data aggregation service, web and mobile accessible surveys, incentives for survey completion and high wear time, available technological support and replacement watches, and newsletters highlighting aggregated statistics of data collected from the smartwatches, reminders to sync smartwatch data, and information about smartwatch features may each have contributed.

### Limitations

This study has several limitations. First, participants volunteered, and therefore the findings are subject to volunteer bias. Recruited physicians tended to be young, female, and White. Smartwatch technology may not be as appealing to other demographic groups, and this warrants further investigation. Second, although this study was conducted at 2 sites, it included only physicians working in an academic medical center. Additional studies are needed to explore the efficacy of smartwatches for physicians who work in other settings. Third, blinding this active intervention would not be feasible, so participants were aware of their assigned study group. Expectancy effects and social desirability bias could have influenced self-reported outcomes. The substantial reductions in burnout and increases in resilience without corresponding improvements in quality of life, depressive symptoms, stress, or sleepiness suggest that the intervention had a targeted effect on specific aspects of well-being. If expectancy alone had driven the results, we would have expected more global improvements across all domains. Fourth, crossover contamination was also possible, especially because smartwatches are ubiquitous; however, statistically and clinically significant differences were observed between the intervention and control arms, suggesting that any contamination that may have occurred was limited and did not substantially affect the outcomes. Fifth, we do not know how or which type of individual behaviors changed due to wearing the smartwatch and having access to one’s physiologic and physical activity data. It is also possible that the structured outreach—including newsletters and adherence support—may have signaled investment in participants’ well-being, contributing to the observed effects. Finally, we did not ask participants in the immediate intervention arm to stop wearing their smartwatch during the second half of the study. Therefore, we were unable to assess postintervention effects or durability of the benefit of having worn a smartwatch if it was no longer used.

## Conclusions

The findings of this randomized clinical trial suggest that wearing a smartwatch can reduce burnout and improve resilience among physicians. Because this intervention was individually focused, it should be considered as an evidence-based strategy to immediately support physicians while other longer-term initiatives address the systemic factors contributing to high work stress among physicians.

## Notes

Supplement 1.
Trial Protocol

Supplement 2. **eTable 1.** Burnout, Quality of Life, Depressive Symptoms, Stress, Sleepiness, and Resilience of Participants at 3 and 6 Months **eTable 2.** Multivariable Model for Emotional Exhaustion and Depersonalization **eTable 3.** Multivariable Model for Quality of Life, Depressive Symptoms, Stress, and Sleepiness **eTable 4.** Multivariable Models for Burnout and Resilience at 9 and 12 Months **eTable 5.** Paired Analysis: Delayed Intervention Cohort, 6 to 12 months **eFigure.** Average Wear Time of Study Participants

Supplement 3.
Data Sharing Statement
