# Supplementary material for: Smartwatch Use and Physician Well-Being: A Randomized Clinical Trial
Source: JAMA Netw Open. 2025 Aug 18;8(8):e2527275. doi: 10.1001/jamanetworkopen.2025.27275 (PMC12362228; doi:10.1001/jamanetworkopen.2025.27275)
Supplement: Supplement 1. — Trial Protocol [file jamanetwopen-e2527275-s001.pdf]

COMIRB Protocol

COLORADO MULTIPLE INSTITUTIONAL REVIEW BOARD
CAMPUS BOX F-490 TELEPHONE: 303-724-1055 Fax: 303-724-0990

Protocol #: 22-0799
Project Title: CUTHRIVE: Physician Burnout Prediction using Garmin Smartwatch
Principal Investigator: Liselotte N. Dyrbye, MD MHPE
Co-Investigator: Pari Thibodeau, Kirsten Black
Version Date: May 15, 2024

Expedited Review
Funding: Physicians' Foundation; Grants & contracts sponsor # 223216

I. Hypotheses and Specific Aims:

We will conduct a randomized controlled trail to evaluate if wearing a Smartwatch improves overall well-being among physicians, and if so, in which dimension of well-being (e.g., fatigue, stress, overall quality of life, burnout). Additionally, we will explore if data from Smartwatches can predict subsequent well-being among physicians.

Study Aims:
1. To determine if wearing a Smartwatch and having access to its physiological data (e.g., sleep, step count, activity, breathing reminders) improves well-being, and if so which well-being dimensions.
2. To determine whether continuous physiological measures (measured from Smartwatches) contain a 'signal' that predicts physician well-being, and if so in which dimensions.

Hypothesis:
1. Physicians with individualized access to their energy levels, oxygen level, respiratory rate, stress levels, sleep patterns, and heart rate will engage more frequently in behaviors that mitigate stress, resulting in improved well-being.
2. Fatigue, stress, and overall quality of life will improve while burnout will be less impacted by wearing a Smartwatch.
3. Physiologic parameters, collected from Smartwatches, will relate to physician's subsequent endorsement of symptoms of fatigue, stress level, and overall quality of life more closely than their measured symptoms of professional burnout.

In this study, physicians will be asked to wear a Smartwatch and answer a survey at baseline, 3-, 6-, 9-, and 12-months. We will determine if physicians who wear a Smartwatch and have access to its data experience greater improvements in their well-being relative to physicians not wearing a Smartwatch (control group). In addition, we will collect data from the Smartwatches to determine if we can utilize this data to predict subsequent level of well-being. We will build the

infrastructure and a HIPAA compliant de-identified dataset which includes (i) continuous physiology measures (from Smartwatches; raw data gathered for every 15-second epoch) and (ii) survey responses. We will use this dataset to develop predictive models for well-being derived from physiologic measures. Prediction models established using the cohort in Arm 1 will be validated in the cohort in Arm 2 after they cross-over (at the 6-month time-point) and begin using the Smartwatch.

II. Background and Significance:

Upwards of 40% of physicians have burnout. Physicians at the front lines of caring for patients with COVID-19 are at highest risk, and at least since 2011 burnout has been more prevalent among physicians than other US workers. Multiple systematic reviews and meta-analyses have concluded clinician burnout impacts quality of care in important ways.[1,2] For example, studies of clinicians have found burnout is independently associated with medical error, medical malpractice suits, poor professionalism, absenteeism, low job performance, less favorable cost conscious attitudes, racial biases, lower competency, job dissatisfaction, patient dissatisfaction ratings across a variety of domains, and longer patient recovery times.[3-23] Physicians with burnout are approximately twice as likely to quit their jobs and reduce clinical hours, resulting in a loss of productivity and access to care.[9,10,24-26] At an organizational level burnout-attributable costs related to job turnover and reduced clinical hours among physicians alone are approximately $7600 per physician each year with approximately $4.6 billion in cost on a national scale.[27] Additionally, studies have also found burnout to be independently associated with sleep impairments, alcohol abuse/dependence, suicidal ideation, and motor vehicle incidents.[8,28-31] Physicians are at increased risk of suicide compared to the U.S. general population.[32,33]

Unfortunately, burnout is underrecognized by those who suffer from it, and it typically goes undetected until physicians' performance or health deteriorates. There is a moral and business case to develop pragmatic ways to identify physicians at risk for burnout.[34-36] In recognition of the broad impact of physician well-being on individuals, healthcare organizations, and society, The National Academy of Medicine's consensus study on clinician burnout (co-authored by PI Dyrbye) "call(ed) upon leaders in health care organizations and health professions, educational institutions, as well as within the government and industry to prioritize, prevent and mitigate burnout and foster professional well-being for the overall health of individuals and the nation." [21]

Despite these recommendations from the National Academy of Medicine, few intervention studies have been conducted to-date involving residents and physicians to guide personal and organizational strategies.[37] In previous randomized controlled studies, funded by the Physicians Foundation, we demonstrated that professional coaching reduced overall burnout and improved quality of life and resilience among physicians.[38,39] It remains unknown, however, if physicians at risk for burnout or poor well-being in other dimensions will recognize it and proactively take steps to engage in professional coaching.

In this proposed study, we plan to conduct a 2-arm randomized controlled trial evaluating the effectiveness of 6 months of wearing a Smartwatch and having access to one's physiologic data to improve well-being among physicians. There is increased ubiquity of wearable technologies such as Smartwatches, that provide quantitative measures of one's physiological functioning (e.g., heartrate, sleep cycles, sleep quality) that may serve as a real-time surveillance mechanism to

forecast impending burnout or changes in other dimensions in well-being, potentially triggering individual intervention before suffering or negative impact to patient care occurs. We anticipate that providing physicians with individualized access to their energy levels, oxygen level, respiratory rate, stress levels, sleep patterns, and heart rate will promote behaviors that mitigate stress, resulting in improved well-being.

Additionally, to preempt burnout among physicians, there is an urgent and unmet need for predicting imminent burnout. The National Academy of Medicine's consensus study on clinician burnout recommended that organizations regularly deploy surveys to measure and monitor physician well-being. Although surveys are an important tool, at best they prompt action in work units where quality of care has already suffered, and turnover or loss of productivity has already occurred due to high levels of burnout. Additionally, individuals are typically not made aware of their own level of distress because of completing an organizational survey. Physiologic parameters, collected from Smartwatches, may be able to predict burnout ahead of its observable manifestations. Such information could help individuals as well as organizations engage in proactive interventions to mitigate risk of future burnout, protecting patients and physicians. Burnout can be addressed and therefore, early identification could have a profound broad impact on physicians, patients, employers, and the global economy (improved productivity, work performance and reduced absenteeism).

III. Preliminary Studies/Progress Report:

Few intervention studies have been conducted to-date involving residents and physicians to guide personal and organizational strategies.[37] In previous randomized controlled studies, funded by the Physicians Foundation, we demonstrated that professional coaching reduced overall burnout and improved quality of life and resilience among physicians.[38,39] It remains unknown, however, if physicians at risk for burnout or poor well-being in other dimensions will recognize it and proactively take steps to engage in professional coaching.

IV. Research Methods

A. Outcome Measure(s):

Overall well-being as measured by the Well-Being Index[40-42]
Overall Burnout, emotional exhaustion, and depersonalization, as measured by the Maslach Burnout Inventory[43]
Quality of life as measured by a Linear Analogue Self-Assessment scale[44]
Depressive symptoms as measured by the PROMIS measure 4a shortform
Stress as measured by the Perceived Stress Scale
Fatigue as measured by the Epworth Sleepiness Scale

B. Description of Population to be Enrolled:

Age range: 19 to 80
Up to 184 subjects enrolled at all sites
Enrollment will not be limited based on sex, gender, race or ethnicity.

Inclusion criteria: Physician (residents/fellows or practicing faculty physician) employed by the University of Colorado School of Medicine or Mayo Clinic with no anticipated departure within 18 months of enrollment. Physicians must be employed full-time or part-time (at 60% FTE or higher) and have an Android or iOS smart phone.
Exclusion criteria: Not in the age range. Not a physician employed by the University of Colorado School of Medicine or Mayo Clinic. Does not own an Android or iOS smart phone. Non-reading subjects. Residents/fellows in their last final year of their respective training program.

Residents/fellows may participate in the study, and can be vulnerable to coercion. They do, however, experience burnout and may benefit from benefit from insight into their well-being and future technology able to predict burnout so that it can be mitigated before it occurs. To protect their welfare, we will inform all participants that their decision to participate in this study is entirely voluntary and will not affect their current or future medical care, education, or training at the organization. Additionally, on the survey they may skip any question they do not feel comfortable answering. Residents / fellows may receive an e-mail invitation to participate or learn about the study from other sources. Participation will be optional.

Our power calculations account for a 15% drop-out rate due to watches getting stolen/damaged. Given the study incentives and enthusiasm of our residents and faculty physicians to address burnout and wear novel technologies, we anticipate dropouts will be minimal. In our previous intervention studies, we have been able to meet recruitment goals with survey completion rates of over 90%.[38,39,45]

C. Study Design and Research Methods

Similar to our previous protocols,[38,39] we will conduct a 2-arm randomized controlled trial evaluating if wearing a Smartwatch and having access to its data improves well-being, and if so which well-being dimensions. Additionally, we will collect continuous physiological measures (measured from Smartwatches) to determine if they contain a ‘signal’ that predicts physician well-being, and if so in which dimensions. Findings will be shared as generalizable knowledge through peer-reviewed publications and presentations.

Administrative records will be used to identify physicians employed by University of Colorado School of Medicine and Mayo Clinic. Participants will be recruited through electronic communications, flyers, and announcements (see scripts). No more than 3 e-mails will be sent. In partnership with leadership, investigators and trained research staff will recruit potential study participants by email using administrative rosters, which contain information on primary work units assigned and email addresses. We will also liaison with management for more informal recruitment efforts. This will include advertising by internet (intranet only), flyers, and by oral presentations at work unit meetings (by invitation only). We will explain the aims of the study and summarize the research procedures. Opportunities to address questions and potential concerns will be provided.

Those are interested in study participation can contact the study team using the contact information listed on advertising materials, or they can scan a QR code or click on a URL link that takes them to a web form (in MS Forms or Qualtrics) that contains information about the study and a screening form. Study coordinators will review the screening form and send an email to

eligible physicians, inviting them to enroll in the study by reading and electronically signing the consent form. (see consent form). If the physician has additional questions, they may contact the PI, co-investigator, or study coordinator. Information collected by the screening form for physicians who opt not to join the study will be deleted.

We anticipate recruitment will take no longer than 3 months.

Once we have recruited the appropriate number of physicians, we will administer the baseline survey. After completion of the baseline survey, Ccnsented physicians will be randomly assigned via computer-generated algorithm to one of two groups: Arm 1 = Smartwatch Intervention; Arm 2 = Control. Randomization will be stratified by gender, specialty, work site, and physician category (resident or physician in-practice) using permuted blocks.

Participants randomized to the Smartwatch Intervention arm will receive a Garmin Smartwatch after enrollment and completion of the baseline survey. Garmin Smartwatches are sleek and provide the broadest range of all-day health monitoring features to keep track of energy levels, pulse oximetry, respiratory rate, stress levels, sleep patterns, and heart rate while operating with 18-hour battery life per charge cycle. Participants will be asked to wear the Garmin watch much as possible during the 12-month study period.

Participants randomized to the Control arm will receive no intervention for the first six months of the study, at as which point they will receive Garmin Smartwatches after completion of the 6 month survey.

Smartwatch Issuance. Study subjects will be issued a Garmin Venu 2 Plus or Venu 3s smartwatch (control group subjects will receive the Venu 3s model). For these procedures, trained research staff will de-identify subjects (subject ID and anonymized number ID maintained by PIs) and enroll them into Garmin Connect (for collecting data from Garmin smartwatches) and Fitabase The study coordinator will work with each subject to identify the best way (time and place) to deliver their smartwatch. At the time of delivery, subjects will also receive a printed manual with step-by-step instructions for setting up linkages with Garmin and Fitabase. Prior to delivery, each subject will also receive an individualized email with an unique link needed for setting up the Fitabase data extractor.

Research staff will train subjects on the use and basic maintenance of the smartwatches. Subjects will be informed that research staff can be contacted any time questions arise about the basic operation and maintenance of the smatwatches. Contact information for research staff will be provided.

Study team will promote engagement through bi-weekly in person visits or email as a way to remind regular synchronization of their watch data or completion of quarterly surveys.

Study team will also disseminate a periodic newsletter upon IRB approval (each time), wherein study progress, aggregate data collected (e.g., cumulative statistics on sleep, hours of data, and wear-time), and sample statistics (e.g., unit-level total numbers) are reported. We will also

feature a participant's experience (e.g., positive experience with watch, or an anecdote) only if they consent to sharing – with the following message below. We will provide the participant the option to not be identified, or if they choose to be identified, we will provide the disclaimer also stated below.

> Dear participants, ***welcome*** to the 1st edition of our newsletters! We want to use this newsletter as a fun way to share ***your*** experiences and thoughts during the study. ***If you have anything to share about your experiences in the study such as fun stories, photos or anecdotes – we want to hear from you!*** *While the newsletter is broadcasted to participants of the study, we cannot guarantee the contents of the newsletter will not be shared to others by participants. If you want to share anything for our newsletters, and we decide to feature it, we will send the final version of the newsletter for your approval before it is released. If you have a story idea, please email it to Kirsten Black (*kirsten.black@cuanschutz.edu*).*

All participants will be asked to complete surveys at baseline and at 3-, 6-, 9- and 12-months (see cover letters). We will use MS Excel for maintaining a list of participants, device serial number and basic information (e.g., department). The quarterly surveys will be collected via Mayo Clinic's Survey Research Center, which stores data from Qualtrics. Trained research staff will follow-up via phone if survey not completed within 7 days of the first alert.

The surveys will include the validated Well-Being Index,[40-42] Maslach Burnout Inventory,[43] Linear Analogue Self-Assessment quality of life scale, [44] Perceived Stress Scale, and Epworth Sleepiness Scale,[46] and Quick Inventory of Depressive Symptoms (QIDS-SR). No instrument or question in this survey is designed to diagnose depression or screen for major depressive disorder or suicidality. Each of these metrics has been validated across a wide range of medical conditions and populations, including physicians. Additional self-reported items will explore workplace factors (e.g., work hours, call frequency, shift work, work location [inpatient, outpatient, surgical], etc.), self-reported medical error, and use of organizational provided stress management and fatigue mitigation strategies. Items can be found in the Survey Instrument document. As a token of appreciation, participants will receive $25 for each survey completed.

Finally, smartwatch data (energy levels, respiratory rate, stress levels, sleep patterns, and heart rate) will be collected via FitaBase - a 3rd party data aggregator which will not collect any PHI. We have already tested and developed a parser to query Garmin's system every 14-days, download the raw data, and process them for analyses by summarizing the sensor features by the minute, hour, day, week or month and store into REDCap database.

During the final quarter of the study, March 1-May 31,2024 participants who wear their watches for an average of 70% of the time will receive a $25 e-gift card. This purpose of this incentive is to maximize data to enhance analysis. The data analytic methods rely on complete data for meaningful predictions and can only handle data missingness up to 30%. The wear time goal reflects the threshold needed for data analysis. Wear time will be measured by the watch based on detected heart rate. Participants will be notified of the incentive through the regular newsletter.

During the final quarter of the study, March 1-May 31,2024 participants who wear their watches for an average of 70% of the time will receive a $25 e-gift card. This purpose of this incentive is to

maximize data to enhance analysis. The data analytic methods rely on complete data for meaningful predictions and can only handle data missingness up to 30%. The wear time goal reflects the threshold needed for data analysis. Wear time will be measured by the watch based on detected heart rate. Participants will be notified of the incentive through the regular newsletter.

As the study progresses, we will collect physiologic data from the Smartwatches and build the data infrastructure (Aim 2), and as data accumulates, develop predictive models for burnout (3) using data from the intervention cohort (development cohort for the prediction model) followed by data from the control cohort (validation cohort). To complete this work, we will leverage expertise in computer engineering, machine learning, and artificial intelligence to develop an analytical framework that combines probabilistic graphical models (PGMs) and multitask learning (MTL) to derive interpretable predictions of burnout.

Study drop out mitigation strategies: .

If the watch is not worn for 7 consecutive days while reporting to work, study staff will ask the study participant about any discomfort related to study participation, and will encourage them to continue to wear the watch to the extent they are comfortable (indicating that we may miss data such as sleep).

We have extensive experience conducting randomized controlled studies and have successful employed these methods in our prior research.

BEHAVIORAL HEALTH RISK MANAGEMENT PLAN
The enrollment for this study is not targeting persons with mental health conditions, and this study is not a clinical intervention study. Nevertheless, parts of the survey will ask respondents to recall unpleasant feelings and symptoms of depression. However, these questions are not diagnostic tools for depression or active intent to harm self/others. Responses to these questions are optional and participants may skip answering these questions if they choose.

Potential participants will be notified in the consent form that: “This study includes the assessment of depression symptoms; however, the study staff will not be actively monitoring for depression, treating depression, or referring you for treatment. If you experience depression, thoughts of suicide, or any other mental health concern during the study you will be responsible for arranging for medical care.”

D. Description, Risks and Justification of Procedures and Data Collection Tools:

We do not foresee any adverse consequences for study participants. They will be asked to complete surveys in addition to wearing Smartwatches and sharing resulting physiologic data. The surveys and physiologic data from Smartwatches will be confidential and private. Database for this study will be password-protected and accessible only by authorized (by the study PI) research staff. All study datasets will be deidentified in accordance with HIPAA guidelines. Therefore, each study participant will be assigned a unique study subject ID number. Data collected from smartwatches and surveys will be linked to each subject ID number. For purposes of achieving the

analytic aims described above, inputs will include subject ID number, subject sex, data collected from smartwatches, and scores on surveys. Subject names, addresses, telephone numbers, or any other data that could serve as identifiers will not be entered into the study databases used for data analysis.

In regards to electronic data, we will use MS Excel for maintaining a list of participants, device serial number and basic information (e.g., department). The quarterly surveys will be collected via Mayo Clinic's Survey Research Center (they will send surveys to participants at both sites), which stores data from Qualtrics. Finally, smartwatch data will be collected via FitaBase - a 3rd party data aggregator which will not collect any PHI.

E. Potential Scientific Problems:

A potential problem is that participants wear the watches <50% of the time. This would render the data less usable for downstream analytics. Hence, we will monitor the smartwatch usage data on FitaBase, and if usage is <70%, we will request the participant to wear the watch as often or seek if there are any possible discomforts from the watches.

F. Data Analysis Plan:

For Aim 1, we will compare changes in outcome variables between Arm 1 (Smartwatch Intervention) and Arm 2 (Control group) at 6 months. Then, we will explore the durability of the benefits of having a Smartwatch for 12 months, by examining levels of well-being among Arm 1 participants at the 6- and 12-month mark. We will use generalized estimating equations to account for the repeated-measures design. Tests will be 2-sided, and we will use the intent-to-treat principle in analyses.

To determine whether continuous physiological measures (measured from Smartwatches) contain a 'signal' that predicts physician well-being, and if so in which dimensions. We will construct Probabilistic Graph Models (PGMs) for low overall well-being (score ≥2), burnout (score of ≥27 on emotional exhaustion scale or score of ≥10 on the depersonalization score), low quality of life (≥ ½ SD below the mean), high stress (score ≥ 17), and high fatigue (score ≥ 11). We will derive dynamics for these dimensions of well-being using dynamic Bayesian networks (DBN). Data from Arm 1 will be used to train MTL models to predict risk of low well-being, burnout, low QOL, high stress, and high fatigue in the next quarter. Models will be tested and retrained using data from subsequent assessments. We will then evaluate the predictions using data from Arm 2 (control). Standard metrics of model performance (e.g., AUC, sensitivity, specificity) will be reported. Predictor variables will be ranked by the relative importance in making predictions, and the top 10 predictors (a heuristic for simplicity in deriving easily interpretable predictions in English sentences) will comprise Core Well-being Factors. We aim to achieve predictive accuracies > 70%.

G. Summarize Knowledge to be Gained:

Knowledge gained from this study include: 1) exploring if wearing a Smartwatch and having access to its physiological data improves well-being, and if so which well-being dimensions; and, 2)

determining whether continuous physiological measures (measured from Smartwatches) contain a ‘signal’ that predicts physician well-being, and if so in which dimensions.

H. COMIRB ADVERTISING COMPONENTS SUBMISSION FORM

FIELD TITLE	FIELD TEXT OPTIONS
**Basic elements:**	**PLEASE NOTE: The word “research” or “study” must appear in every ad. All advertisements must include the COMIRB # of the protocol.**<br><br>**The name of the PI, identification of the university, and identification of the study sponsor may be included.**
**Study Title or Ad Header:** *(for studies with lengthy titles, it is acceptable and appropriate to abbreviate the title)*	• Smartwatch Study for Physicians<br>• Smartwatch and Physician Well-Being<br>• Smartwatch and Physician Well-Being: Are Wearables Part of the Solution?
**Purpose of the Research Study:** *(recommend using the word "research")*	• The purpose of the study is to determine if wearing a Smartwatch and having access to its data improves physician well-being in any dimension. A secondary purpose is to determine whether data recorded from Smartwatches contain a ‘signal’ that predicts physician well-being.
**Main Procedures Involved:**	• Physicians in this study will be asked to wear a provided **Smartwatch** and answer a survey at baseline, 3-, 6-, 9- and 12-months.<br>• If you are interested in a <u>free, Garmin Smartwatch</u> consider enrolling in the **Physician Smartwatch** research study. In addition to wearing the watch, participating physicians will be asked to complete surveys at baseline, 3-, 6-, 9- and 12-months
**Main Inclusion/Exclusion Criteria** *(use wording as it will appear in ad)***:**<br><br>**Note: Do not list** **'**English-speaking' as an eligibility requirement	• You must be a physician employed by the University of Colorado School of Medicine or Mayo Clinic to participate. You must also own an Android or iOS smart phone and be able to see the output from the Smartwatch.<br>• To be eligible for the research study, you need to be a physician (resident and fellows also invited!), own an Android or iOS smart phone, and be able to see the output from the Smartwatch
**Duration of Participation:**	The study will last 12 months.<br>• The study will begin in 2022 and last 12 months

**Compensation:**	• Compensation Not Provided. However, you may choose to keep the Smartwatch.<br>• All participants will receive a free Garmin Smartwatch (typically $450value).
**Contact information language:**	• Interested physicians at the University of Colorado School of Medicine / CU Medicine and Mayo Clinic are invited to contact any of the following for further information:<br>"[name]" at"[phone]" or "[e-mail]”<br>"[name]" at"[phone]" or "[e-mail]”<br>"[name]" at"[phone]" or "[e-mail]”
**Other ad text/features (e.g.: contact tear-off tabs, linked websites)**	Looking for a way to improve your health and well-being? If so, a Smartwatch may be just what you need!<br><br>A Smartwatch can help you:<br>• Track of energy levels, pulse oximetry, respiratory rate, stress levels, and sleep pattern<br>• Become more self-aware<br>• Increase your motivation to take action<br>• Enhance your well-being

I. Cover letter for surveys

Dear <name of physician>,

Thank you for agreeing to participate in the Smartwatch study. We would appreciate your assistance and ask that you take 15 minutes to complete the <baseline / 3 month / 6 month / 12 month> survey. Although we hope you will answer all questions, you can skip any questions you choose not to answer.

Click on the following link or type the link in your browser to complete the survey:__________ You must complete the survey from the link sent directly to you. As a token of appreciation, participants will receive $25 gift card for each survey completed.

Your opinion is important to us. Preserving confidentiality is essential in this study. Researchers will not have access to individual responses on survey questions used in this study.

Please continue to wear your watch through the end of June. We will send you one final newsletter announcing when the study team has stopped monitoring your smartwatch data and showing you the final dashboard of data collection.

If you have any questions or concerns, please feel free to e-mail SmartWatch@olucdenver.onmicrosoft.com or reach out to Kirsten Black

(kirsten.black@cuanschutz.edu) or Lotte Dyrbye, M.D. (liselotte.dyrbye@cuanschutz.edu). If you encounter problems in filling out the survey itself, please contact Mayo Clinic Survey Research Center (surveyresearchcenter@mayo.edu).

With Regards,

Lotte Dyrbye, MD, MHPE
Professor of Medicine
Chief Well-being Officer and Senior Associate Dean of Faculty
University of Colorado School of Medicine

Cover letter for 6 month survey sent to the Control Group

Dear <name of physician>,

Thank you for agreeing to participate in the Smartwatch study. We would appreciate your assistance and ask that you take 15 minutes to complete the 6 month survey. Although we hope you will answer all questions, you can skip any questions you choose not to answer.

Click on the following link or type the link in your browser to complete the survey:_________ You must complete the survey from the link sent directly to you. As a token of appreciation, participants will receive $25 gift card for each survey completed. Additionally, after completing the 6 month survey, we will coordinate with you to distribute your smartwatch.

Your opinion is important to us. Preserving confidentiality is essential in this study. Researchers will not have access to individual responses on survey questions used in this study.

If you have any questions or concerns, please feel free to e-mail SmartWatch@olucdenver.onmicrosoft.com or reach out to Kirsten Black (kirsten.black@cuanschutz.edu) or Lotte Dyrbye, M.D. (liselotte.dyrbye@cuanschutz.edu). If you encounter problems in filling out the survey itself, please contact Mayo Clinic Survey Research Center (surveyresearchcenter@mayo.edu).

With Regards,

Lotte Dyrbye, MD, MHPE
Professor of Medicine
Chief Well-being Officer and Senior Associate Dean of Faculty
University of Colorado School of Medicine

J. Survey Instrument

(Note: The survey will be converted to electronic format and headers will be removed.)

A. PROFESSIONAL CHARACTERISTICS AND DEMOGRAPHICS

1A. Which category best describes your practice:

a. anesthesiology
b. dermatology
c. emergency medicine
d. family medicine
e. internal medicine or related specialty
f. radiology
g. neurology
h. obstetrics and gynecology
i. ophthalmology
j. pathology
k. pediatrics or related specialty
l. physical medicine and rehabilitation
m. preventive medicine, occupational medicine, or environmental medicine
n. psychiatry
o. radiation oncology
p. surgical specialty
q. other, please specify below: ______________

1B. Which category best describes your surgical practice (for those who answer "surgical specialty" for question 1A)

a. Breast surgery
b. Cardio-thoracic Surgery
c. Colorectal Surgery
d. General Surgery
e. Neurologic Surgery
f. Oncologic surgery
g. Orthopedic Surgery
h. Otolaryngology
i. Pediatric Surgery
j. Plastic Surgery
k. Transplant Surgery
l. Trauma Surgery
m. Urologic Surgery
n. Vascular Surgery
o. Other, please specify below: _______________

1C. Which category best describes your practice (for those who answer either "internal medicine or related specialty" for question 1A)

1. Allergy/immunology
2. Cardiology
3. Endocrinology
4. Gastroenterology and hepatology

5. General internal medicine – primarily outpatient
6. General internal medicine – primarily inpatient (hospitalist)
7. Geriatrics
8. Hematology/Oncology
9. Infectious disease
10. Nephrology
11. Pulmonary/critical care medicine – without critical care medicine
12. Pulmonary/critical care medicine – with critical care medicine
13. Rheumatology
14. Other, please specify below: _____

1D. Which category best describes your practice (for those who answer “pediatrics or related specialty” for question 1A)

a. Adolescent medicine
b. Allergy/immunology
c. Cardiology
d. Critical care medicine
e. Developmental/behavioral pediatrics
f. Emergency medicine
g. Endocrinology
h. Gastroenterology and hepatology
i. General Pediatrics
j. Genetics
k. Hematology/Oncology
l. Infectious disease
m. Neonatology
n. Nephrology
o. Pulmonary medicine
p. Rheumatology
q. Other, please specify: _____

2. In a typical week:

a. how many nights are you on call (0-7)?______ (drop down menu 0-7)

b. how many hours do you work?______ (enter #)

3. A full-time equivalent (FTE) is a unit that indicates the workload of a person in a way that makes it comparable across contexts. An FTE of 100% is considered full-time, while an FTE of 50% signals half time.

According to your organization or practice group, what is your current FTE (range 0-100%)? ____

4. What is your % FTE dedicated to direct patient care activities?

a. less than 10%
b. 10-24%
c.25-49%

d. 50-74%
e. 75-99%
f. 100%

5. Please indicate your current role:
   a. Physician in-practice (faculty, finished with residency/fellowship)
   b. Intern or Resident
   c. Fellow
6. Please indicate your level of training (if answered 5 b or c, resident/fellow)
   a. PGY-1
   b. PGY-2
   c. PGY-3
   d. PGY-4
   e. PGY-5
   f. PGY-6
   g. More than PGY-6

7. (If answered 5a) How many years have you been in practice (i.e., out of residency/fellowship training)? ______ (enter #)

8. What is your age: ___ (enter #)

9. What is your gender
   a. male
   b. female
   c. other

10. What is your racial background (Mark all that apply.)
   a. White or Caucasian
   b. Black or African American
   c. Asian
   d. American Indian or Alaska Native
   e. Pacific Islander or Native Hawaiian
   d. Other, please specify: ___________

11. Are you of Hispanic or Latino origin?
   a. No
   b. Yes
   c. I don't know __________

12. What is your current relationship status:
   a. Single
   b. Married
   c. Partnered
   d. Widowed/widower

B. QUALITY OF LIFE (LASA)

1. How would you describe your overall quality of life during the past week, including today (0 = As bad as it can be, 10 = As good as it can be)?

0 1 2 3 4 5 6 7 8 9 10

B. BURNOUT (MBI)

Used under license from Mind Garden, Inc.

F. RESILIENCE

Please select the option below that best indicates how much you agree with the following statements as they apply to you over the last month. If a particular situation has not occurred recently, answer according to how you think you would have felt.

1. I am able to adapt to change
2. I can deal with whatever comes
3. I can see the humorous side of things
4. I believe coping with stress strengthens
5. I tend to bounce back after illness, injury, or other hardship
6. I believe I can achieve your goals
7. When under pressure I can focus and think clearly
8. I am not easily discouraged by failure
9. I think of myself as a strong person
10. I can handle unpleasant feelings

Not true at all (0), rarely true (1), sometimes true (2), often true (3), and true nearly all of the time (4)

G. PERCEIVED STRESS SCALE

The questions in this scale ask about your feelings and thoughts
during the last month. In each case, you will be asked to indicate how often you felt or thought a certain way. Although some of the questions are similar, there are differences between them and you should treat each one as a separate question. The best approach is to answer fairly quickly. That is, don't try to count up the number of times you felt a particular way; rather indicate the alternative that seems like a reasonable estimate.

For each question choose from the following: 0 = never, 1 = almost never, 2 = sometimes, 3 = fairly often, 4 = very often

l. In the last month, how often have you been upset because of something that happened unexpectedly?
2. In the last month, how often have you felt that you were unable to control the important things in your life?
3. In the last month, how often have you felt nervous and stressed?

4. In the last month, how often have you felt confident about your ability to handle your personal problems?
5. In the last month, how often have you felt that things were going your way?
6. In the last month, how often have you found that you could not cope with all the things that you had to do?
7. In the last month, how often have you been able to control irritations in your life?
8. In the last month, how often have you felt that you were on top of things?
9. In the last month, how often have you been angered because of things that happened that were outside of your control?
10. In the last month, how often have you felt difficulties were piling up so high that you could not overcome them?

H. EPWORTH SLEEPINESS SCALE

How likely are you to doze off or fall asleep in the following situations? You should rate your chances of dozing off, not just feeling tired. Even if you have not done some of these things recently try to determine how they would have affected you. For each situation, decide whether or not you would have:

· No chance of dozing =0
· Slight chance of dozing =1
· Moderate chance of dozing =2
· High chance of dozing =3

Sitting and reading ·
Watching TV ·
Sitting inactive in a public place (e.g., a theater or a meeting)
As a passenger in a car for an hour without a break
Lying down to rest in the afternoon when circumstances permit
Sitting and talking to someone ·
Sitting quietly after a lunch without alcohol ·
In a car, while stopped for a few minute

I. WELL-BEING INDEX

- Access the WBI for research (https://www.mywellbeingindex.org/research-request/)

J. PROMIS SHORT FORM 4A. EMOTIONAL DISTRESS – DEPRESSIVE SYMPTOMS

In the past 7 days how often have you felt

a) Worthless
b) Helpless
c) Depressed
d) Hopeless

Response options: never, rarely, sometimes, often, always

K. OTHER

1. Are you concerned you have made a major medical error in the last 3 months?
   a. Yes
   b. No

2. 3 and 6 month control group only:

1. Are you currently wearing a Smartwatch (e.g., Garmin, Apple watch) or some kind of tracker device (e.g., Fitbit Charge, WHOOP, Basis B1 (n=1)26, Empatica E4, Microsoft Smart Band 2 HealthPatch) ?
   a. Yes
   b. No

References:

1. Dewa CS, Loong D, Bonato S, Trojanowski L. The relationship between physician burnout and quality of healthcare in terms of safety and acceptability: A systematic review. BMJ Open 2017;7(6) (Review). DOI: 10.1136/bmjopen-2016-015141.
2. Salyers MP, Bonfils KA, Luther L, et al. The Relationship Between Professional Burnout and Quality and Safety in Healthcare: A Meta-Analysis. J Gen Intern Med 2017;32(4):475-482.
3. Dyrbye L, Herrin J, West CP, et al. Association of Racial Bias With Burnout Among Resident Physicians. JAMA Network Open 2019;2(7):e197457-e197457. DOI: 10.1001/jamanetworkopen.2019.7457.
4. Dyrbye LN, Burke SE, Hardeman RR, et al. Association of clinical specialty with symptoms of burnout and career choice regret among us resident physicians. JAMA 2018;320(11):1114-1130. DOI: 10.1001/jama.2018.12615.
5. Dyrbye LN, West CP, Leep Hunderfund AN, et al. Relationship Between Burnout, Professional Behaviors, and Cost-conscious Attitudes Among US Physicians. J Gen Intern Med 2020;35(5):1465-1476.
6. Shanafelt TD, Balch CM, Bechamps G, et al. Burnout and medical errors among American surgeons. Ann Surg 2010;251(6):995-1000. (In English). DOI: https://dx.doi.org/10.1097/SLA.0b013e3181bfdab3.
7. Shanafelt TD, Balch CM, Bechamps GJ, et al. Burnout and career satisfaction among American surgeons. Ann Surg 2009;250(3):463-71. (In eng). DOI: 10.1097/SLA.0b013e3181ac4dfd.
8. Shanafelt TD, Balch CM, Dyrbye LN, et al. Suicidal ideation among American surgeons. Arch Surg 2011;146(1):54-62.
9. Shanafelt TD, Dyrbye LN, West CP, Sinsky CA. Potential Impact of Burnout on the US Physician Workforce. Mayo Clin Proc 2016;91(11):1667-1668. (In eng). DOI: 10.1016/j.mayocp.2016.08.016.
10. Windover AK, Martinez K, Mercer MB, Neuendorf K, Boissy A, Rothberg MB. Correlates and outcomes of physician burnout within a large academic medical center. JAMA Intern Med 2018;178(6):856-858. (Letter). DOI: 10.1001/jamainternmed.2018.0019.
11. West CP, Dyrbye LN, Shanafelt TD. Physician burnout: contributors, consequences and solutions. Journal of internal medicine 2018;283(6):516-529. (In eng). DOI: 10.1111/joim.12752.

12. West CP, Tan AD, Habermann TM, Sloan JA, Shanafelt TD. Association of resident fatigue and distress with perceived medical errors. JAMA 2009;302(12):1294-300. (In eng). DOI: 10.1001/jama.2009.1389.
13. Tawfik DS, Profit J, Morgenthaler TI, et al. Physician Burnout, Well-being, and Work Unit Safety Grades in Relationship to Reported Medical Errors. Mayo Clinic Proceedings 2018 (Article in Press). DOI: 10.1016/j.mayocp.2018.05.014.
14. Balch CM, Oreskovich MR, Dyrbye LN, et al. Personal consequences of malpractice lawsuits on American surgeons. J Am Coll Surg 2011;213(5):657-67. (In eng). DOI: 10.1016/j.jamcollsurg.2011.08.005.
15. Lu DW, Dresden SM, Mark Courtney D, Salzman DH. An Investigation of the Relationship Between Emergency Medicine Trainee Burnout and Clinical Performance in a High-fidelity Simulation Environment. AEM Educ Train 2017;1(1):55-59. (In eng). DOI: 10.1002/aet2.10004.
16. Leiter MP, Harvie P, Frizzell C. The correspondence of patient satisfaction and nurse burnout. Soc Sci Med 1998;47(10):1611-7. (Research Support, Non-U.S. Gov't)
17. Vahey DC, Aiken LH, Sloane DM, Clarke SP, Vargas D. Nurse burnout and patient satisfaction. Med Care 2004;42(2 Suppl):II57-66. (Research Support, Non-U.S. Gov't
Research Support, U.S. Gov't, P.H.S.) (In English)
18. McHugh MD, Kutney-Lee A, Cimiotti JP, Sloane DM, Aiken LH. Nurses' widespread job dissatisfaction, burnout, and frustration with health benefits signal problems for patient care. Health Aff 2011;30(2):202-10. (Research Support, N.I.H., Extramural
Research Support, Non-U.S. Gov't) (In English). DOI: https://dx.doi.org/10.1377/hlthaff.2010.0100.
19. Halbesleben JR, Rathert C. Linking physician burnout and patient outcomes: exploring the dyadic relationship between physicians and patients. Health Care Manage Rev 2008;33(1):29-39. (In eng). DOI: 10.1097/01.Hmr.0000304493.87898.72.
20. Dyrbye LN, Shanafelt TD, Johnson PO, Johnson LA, Satele D, West CP. A cross-sectional study exploring the relationship between burnout, absenteeism, and job performance among American nurses. BMC Nursing 2019;18(1):57. DOI: 10.1186/s12912-019-0382-7.
21. National Academies of Sciences Engineering and Medicine. Taking Action Against Clinician Burnout: A Systems Approach to Professional Well-Being. Washington, DC: The National Academies Press, 2019.
22. West CP, Shanafelt TD, Kolars JC. Quality of life, burnout, educational debt, and medical knowledge among internal medicine residents. JAMA 2011;306(9):952-960. (Article). DOI: 10.1001/jama.2011.1247.
23. West CP, Huschka MM, Novotny PJ, et al. Association of perceived medical errors with resident distress and empathy: a prospective longitudinal study. JAMA 2006;296(9):1071-8. (In eng). DOI: 10.1001/jama.296.9.1071.
24. Hamidi MS, Bohman B, Sandborg C, et al. Estimating institutional physician turnover attributable to self-reported burnout and associated financial burden: a case study. BMC Health Serv Res 2018;18(1):851. (In eng). DOI: 10.1186/s12913-018-3663-z.
25. Willard-Grace R, Knox M, Huang B, Hammer H, Kivlahan C, Grumbach K. Burnout and Health Care Workforce Turnover. Ann Fam Med 2019;17(1):36-41. (In eng). DOI: 10.1370/afm.2338.
26. Shanafelt TD, Mungo M, Schmitgen J, et al. Longitudinal Study Evaluating the Association Between Physician Burnout and Changes in Professional Work Effort. Mayo Clin Proc 2016;91(4):422-31. (Research Support, Non-U.S. Gov't) (http://ovidsp.ovid.com/ovidweb.cgi?T=JS&CSC=Y&NEWS=N&PAGE=fulltext&D=medl&AN=27046522).
27. Han S, Shanafelt TD, Sinsky CA, et al. Estimating the Attributable Cost of Physician Burnout in the United StatesCost of Physician Burnout. Ann Intern Med 2019;170(11):784-790. DOI: 10.7326/m18-1422.
28. Oreskovich M, Kaups K, Balch C, et al. The prevalence of alcohol use disorders among american surgeons. Arch Surg 2011;147(2):168-174.
29. Dyrbye LN, Thomas MR, Massie FS, et al. Burnout and suicidal ideation among US medical students. Ann Intern Med 2008;149:334.

30. West CP, Tan AD, Shanafelt TD. Association of resident fatigue and distress with occupational blood and body fluid exposures and motor vehicle incidents. Mayo Clinic Proc 2012;87(12):1138-44. (Research Support, Non-U.S. Gov't) (In eng). DOI: 10.1016/j.mayocp.2012.07.021.
31. Trockel MT, Menon NK, Rowe SG, et al. Assessment of Physician Sleep and Wellness, Burnout, and Clinically Significant Medical Errors. JAMA Netw Open 2020;3(12):e2028111. (In eng). DOI: 10.1001/jamanetworkopen.2020.28111.
32. Center C, Davis M, Detre T, et al. Confronting depression and suicide in physicians: a consensus statement. JAMA 2003;289(23):3161-6. (In eng). DOI: 10.1001/jama.289.23.3161.
33. Davidson JE, Proudfoot J, Lee K, Terterian G, Zisook S. A Longitudinal Analysis of Nurse Suicide in the United States (2005–2016) With Recommendations for Action. Worldviews Evid Based Nurs 2020;17(1):6-15. DOI: 10.1111/wvn.12419.
34. Dzau VJ, Kirch DG, Nasca TJ. To Care Is Human - Collectively Confronting the Clinician-Burnout Crisis. N Engl J Med 2018;378(4):312-314. (In eng). DOI: 10.1056/NEJMp1715127.
35. Dyrbye LN, T.D. Shanafelt, C.A. Sinsky, P.F. Cipriano, J. Bhatt, A. Ommaya, C.P. West, and D. Meyers. Burnout among health care professionals: A call to explore and address this underrecognized threat to safe, high-quality care. 2017:National Academy of Medicine, Washington DC.
36. Dyrbye LN, Shanafelt TD. Physician Burnout. A potential threat to successful health care reform. JAMA 2011;305(19):2009-2010.
37. West CP, Dyrbye LN, Erwin PJ, Shanafelt TD. Interventions to prevent and reduce physician burnout: a systematic review and meta-analysis. Lancet 2016;388:2272-81. DOI: 10.1016/s0140-6736(16)31279-x.
38. Dyrbye LN, Shanafelt TD, Gill PR, Satele DV, West CP. Effect of a Professional Coaching Intervention on the Well-being and Distress of Physicians: A Pilot Randomized Clinical Trial. JAMA Intern Med 2019;179(10):1406-1414. DOI: 10.1001/jamainternmed.2019.2425.
39. Dyrbye L.N., Gill P, Satele D, West CP. Professional Coaching and Surgeon Well-Being. A Randomized Controlled Trial. Ann Surg Revision invited.
40. Dyrbye LN, Satele D, Shanafelt T. Ability of a 9-Item Well-Being Index to Identify Distress and Stratify Quality of Life in US Workers. J Occup Environ Med 2016;58(8):810-7.
41. Dyrbye LN, Satele D, Sloan J, Shanafelt TD. Utility of a brief screening tool to identify physicians in distress. J Gen Intern Med 2013;28(3):421-7. (Evaluation Studies Research Support, Non-U.S. Gov't) (In eng). DOI: 10.1007/s11606-012-2252-9.
42. Dyrbye LN, Satele D, Sloan J, Shanafelt TD. Ability of the Physician Well-Being Index to identify residents in distress. J Grad Med Educ 2014;6(1):78-84.
43. Maslach C, Jackson SE, Leiter MP. Maslach Burnout Inventory. 4th ed2016.
44. Gudex C, Dolan P, Kind P, Williams A. Health state valuations from the general public using the visual analogue scale. Qual Life Res 1996;5(6):521-531.
45. West C, Dyrbye L, Rabatin J, et al. Intervention to promote physician well-being, job satisfaction, and professionalism: a randomized clinical trial. JAMA Intern Med 2014;174(4):527-533.
46. Johns MW. Reliability and factor analysis of the Epworth Sleepiness Scale. Sleep Med 1992;15(4):376-81.
