# Supplementary material for: Smartwatch Use and Physician Well-Being: A Randomized Clinical Trial
Source: JAMA Netw Open. 2025 Aug 18;8(8):e2527275. doi: 10.1001/jamanetworkopen.2025.27275 (PMC12362228; doi:10.1001/jamanetworkopen.2025.27275)
Supplement: Supplement 2. — eTable 1. Burnout, Quality of Life, Depressive Symptoms, Stress, Sleepiness, and Resilience of Participants at 3 and 6 Months eTable 2. Multivariable Model for Emotional Exhaustion and Depersonalization eTable 3. Multivariable Model for Quality of Life, Depressive Symptoms, Stress, and Sleepiness eTable 4. Multivariable Models for Burnout and Resilience at 9 and 12 Months eTable 5. Paired Analysis: Delayed Intervention Cohort, 6 to 12 months eFigure. Average Wear Time of Study Participants [file jamanetwopen-e2527275-s002.pdf]

Supplemental Online Content

Dyrbye LN, West CP, Wilton AR, Satele DV, Athreya AP. Smartwatch use and physician well-being: a randomized clinical trial. *JAMA Netw Open*. 2025;8(8):e2527275. doi:10.1001/jamanetworkopen.2025.27275

eTable 1. Burnout, Quality of Life, Depressive Symptoms, Stress, Sleepiness, and Resilience of Participants at 3 and 6 Months

eTable 2. Multivariable Model for Emotional Exhaustion and Depersonalization

eTable 3. Multivariable Model for Quality of Life, Depressive Symptoms, Stress, and Sleepiness

eTable 4. Multivariable Models for Burnout and Resilience at 9 and 12 Months

eTable 5. Paired Analysis: Delayed Intervention Cohort, 6 to 12 months

eFigure. Average Wear Time of Study Participants

This supplemental material has been provided by the authors to give readers additional information about their work.

© 2025 Dyrbye LN et al. *JAMA Network Open*.

eTable 1. Burnout, Quality of Life, Depressive Symptoms, Stress, Sleepiness, and Resilience of Participants at 3 and 6 Months

	3 Months			6 Months		
	Immediate Arm (n = 86)	Control Arm (n = 88)	P-value	Immediate Arm (n = 85)	Control Arm (n = 91)	P-value
Burnout						
Emotional Exhaustion[1]						
Mean (SD)	22.0 (12.4)	22.6 (11.1)	0.70	20.8 (11.5)	22.1 (10.9)	0.60
High, n (%)			0.37			0.88
Yes	34 (39.5%)	29 (33.0%)		29 (34.1%)	32 (35.2%)	
No	52 (60.5%)	59 (67.0%)		56 (65.9%)	59 (64.8%)	
Depersonalization[2]						
Mean (SD)	7.1 (6.0)	7.9 (6.1)	0.36	6.6 (5.8)	8.0 (6.3)	0.12
High, n (%)			0.55			0.11
Yes	30 (34.9%)	27 (30.7%)		24 (28.2%)	36 (39.6%)	
No	56 (65.1%)	61 (69.3%)		61 (71.8%)	55 (60.4%)	
Overall burnout, n (%)[3]			0.55			0.21
Yes	40 (46.5%)	37 (42.0%)		35 (41.2%)	46 (50.5%)	
No	46 (53.5%)	51 (58.0%)		50 (58.8%)	45 (49.5%)	
Resilience, Mean (SD)[4]	32.0 (5.5)	28.3 (5.4)	<0.001	31.9 (5.0)	29.5 (6.2)	0.01
Quality of life, Mean (SD)[5]	7.0 (1.9)	6.9 (1.7)	0.50	7.0 (1.8)	6.9 (1.7)	0.95
Depressive Symptoms, Mean (SD) [6]	5.8 (2.8)	6.3 (2.8)	0.17	5.9 (2.6)	5.7 (2.5)	0.89
Stress, Mean (SD)[7]	14.9 (6.6)	16.8 (5.7)	0.03	14.5 (6.3)	15.5 (6.0)	0.22
Sleepiness, Mean (SD)[8]	6.5 (4.2)	6.1 (4.2)	0.38	6.2 (4.4)	5.7 (4.2)	0.39

[1] The score range is 0-54; higher score indicates greater burnout symptoms. High emotional exhaustion defined by score of ≥27 on the emotional exhaustion subscale.
[2] The score range is 0-30; higher score indicates greater burnout symptoms. High depersonalization defined by score of ≥10 on the depersonalization subscale.
[3] Positive for symptoms of overall burnout if had a high score (≥27) on the emotional exhaustion and or high score (≥10) on the depersonalization subscale.
[4] The score range is 0-40, with higher scores suggesting greater resilience. Resilience was measured using the 10-item Connor-Davidson Resilience Scale.
[5] The score range is 0-10, with higher scores indicating better quality of life.

© 2025 Dyrbye LN et al. *JAMA Network Open*.

[6] The score range is 4-20, with higher scores suggesting greater depressive symptoms. Depressive symptoms was measured using the PROMIS 4a shortform.
[7] The score range is 0-40, with higher scores suggesting more stress. Stress was measured using the 10 item Perceived Stress Scale.
[8] The score range is 0-24, with higher scores suggesting more sleepiness. Sleepiness was measured using the Epworth Sleepiness Scale.

© 2025 Dyrbye LN et al. *JAMA Network Open*.

eTable 2. Multivariable Model for Emotional Exhaustion and Depersonalization

	3 months			6 months		
	PE[1] (95% CI)	Class p-value	Overall p-value	PE (95% CI)	Class p-value	Overall p-value
Emotional Exhaustion						
Baseline emotional exhaustion	0.86 (0.77, 0.95)		<.001	0.825 (0.74, 0.91)		<.001
Intervention (vs. control)	0.37 (-1.64, 2.38)		0.72	-0.20 (-2.09, 1.68)		0.83
Attending (vs. resident/fellow)	0.96 (-1.91, 3.83)		0.51	-1.44 (-4.15, 1.27)		0.30
Colorado (vs. Minnesota)	-0.62 (-2.71, 1.48)		0.56	-0.87 (-2.84, 1.09)		0.38
Specialty (vs. Primary Care)			0.48			0.71
Non-Primary Care	-0.81 (-3.30, 1.68)	0.52		-0.92 (-3.26, 1.42)	0.44	
Surgical Field	-1.90 (-5.00, 1.19)	0.23		-1.01 (-3.90, 1.88)	0.49	
Work hours per week (for each additional hour)	0.03 (-0.06, 0.12)		0.46	-0.02 (-0.10, 0.07)		0.70
Age in years (for each additional year)	0.09 (-0.06, 0.24)		0.22	0.06 (-0.08, 0.20)		0.40
Female (vs. male)	0.64 (-1.42, 2.69)		0.54	-0.10 (-2.03, 1.83)		0.92
Relationship status (vs. single)			0.16			0.01
Married	0.95 (-2.71, 4.61)	0.61		2.85 (-0.54, 6.24)	0.10	
Partnered	3.62 (-0.60, 7.83)	0.09		6.33 (2.42, 10.25)	0.00	
White or Caucasian (vs. all others)	-0.38 (-2.97, 2.21)		0.77	-0.48 (-2.93, 1.96)		0.70
Not Hispanic or Latino (vs. Hispanic or Latino)	2.77 (-0.60, 6.13)		0.11	-1.37 (-4.54, 1.80)		0.40
Depersonalization						
Baseline depersonalization	0.73 (0.64, 0.82)		<.001	0.71 (0.62, 0.80)		<.001
Intervention (vs. control)	-0.55 (-1.62, 0.52)		0.31	-1.07 (-2.23, 0.09)		0.07
Attending (vs. resident/fellow)	-0.31 (-1.84, 1.23)		0.69	-0.96 (-2.63, 0.71)		0.26
Colorado (vs. Minnesota)	0.23 (-0.90, 1.35)		0.69	-0.29 (-1.49, 0.93)		0.64
Specialty (vs. Primary Care)			0.15			0.59
Non-Primary Care	-0.07 (-1.41, 1.27)	0.92		-0.40 (-1.86, 1.05)	0.58	
Surgical Field	-1.43 (-3.11, 0.26)	0.10		-0.94 (-2.75, 0.87)	0.31	
Work hours per week (for each additional hour)	0.02 (-0.02, 0.07)		0.33	0.02 (-0.03, 0.07)		0.50
Age in years (for each additional year)	0.05 (-0.03, 0.13)		0.22	0.02 (-0.06, 0.11)		0.63
Female (vs. male)	1.12 (0.04, 2.21)		0.04	0.20 (-0.98, 1.37)		0.74
Relationship status (vs. single)			0.27			0.10

© 2025 Dyrbye LN et al. *JAMA Network Open*.

Married	-0.32 (-2.28, 1.64)	0.75		0.44 (-1.65, 2.53)	0.68	
Partnered	1.05 (-1.21, 3.31)	0.36		2.20 (-0.22, 4.61)	0.08	
White or Caucasian (vs. all others)	-0.94 (-2.32, 0.45)		0.18	-0.29 (-1.79, 1.22)		0.71
Not Hispanic or Latino (vs. Hispanic or Latino)	0.03 (-1.78, 1.83)		0.98	-1.36 (-3.32, 0.60)		0.17

[1] PE = parameter estimate for relevant score

© 2025 Dyrbye LN et al. *JAMA Network Open*.

eTable 3. Multivariable Model for Quality of Life, Depressive Symptoms, Stress, and Sleepiness

	3 months			6 months		
	PE[1] (95% CI)	Class p-value	Overall p-value	PE (95% CI)	Class p-value	Overall p-value
Quality of Life (for each 1 point higher)						
Baseline Quality of Life	0.54 (0.37, 0.72)		<.001	0.54 (0.37, 0.71)		<.001
Intervention (vs. control)	-0.06 (-0.55, 0.43)		0.81	0.13 (-0.35, 0.61)		0.59
Attending (vs. resident/fellow)	-0.56 (-1.26, 0.15)		0.12	-0.39 (-1.08, 0.29)		0.26
Colorado (vs. Minnesota)	-0.07 (-0.59, 0.44)		0.78	-0.15 (-0.65, 0.34)		0.55
Specialty (vs. Primary Care)			0.32			0.51
Non-Primary Care	-0.38 (-0.99, 0.23)	0.22		-0.20 (-0.79, 0.40)	0.52	
Surgical Field	-0.54 (-1.29, 0.21)	0.16		0.16 (-0.56, 0.88)	0.66	
Work hours per week (for each additional hour)	-0.001 (-0.02, 0.02)		0.91	-0.01 (-0.03, 0.01)		0.39
Age in years (for each additional year)	0.02 (-0.02, 0.05)		0.30	0.03 (-0.01, 0.06)		0.10
Female (vs. male)	-0.28 (-0.77, 0.22)		0.27	-0.24 (-0.72, 0.24)		0.32
Relationship status (vs. single)			0.32			0.95
Married	-0.007 (-0.90, 0.89)	0.99		0.12 (-0.74, 0.98)	0.79	
Partnered	-0.58 (-1.62, 0.46)	0.27		0.04 (-0.96, 1.04)	0.93	
White or Caucasian (vs. all others)	0.16 (-0.48, 0.81)		0.62	0.02 (-0.61, 0.66)		0.94
Not Hispanic or Latino (vs. Hispanic or Latino)	-0.36 (-1.19, 0.46)		0.39	-0.68 (-1.48, 0.12)		0.10
Depressive symptoms (for each 1 point higher)						
Baseline depression	0.51 (0.38, 0.64)		<.001	0.555 (0.44, 0.67)		<.001
Intervention (vs. control)	-0.24 (-0.93, 0.46)		0.50	0.26 (-0.35, 0.86)		0.41
Attending (vs. resident/fellow)	-0.05 (-1.04, 0.95)		0.93	-0.001 (-0.87, 0.87)		1.00
Colorado (vs. Minnesota)	0.60 (-0.12, 1.32)		0.10	0.05 (-0.57, 0.67)		0.87
Specialty (vs. Primary Care)			0.78			0.49
Non-Primary Care	-0.22 (-1.08, 0.65)	0.62		-0.28 (-1.03, 0.47)	0.46	
Surgical Field	-0.38 (-1.45, 0.70)	0.49		0.16 (-0.76, 1.08)	0.73	
Work hours per week (for each additional hour)	0.02 (-0.01, 0.05)		0.17	0.01 (-0.01, 0.04)		0.33
Age in years (for each additional year)	0.009 (-0.04, 0.06)		0.73	-0.000 (-0.04, 0.04)		0.99
Female (vs. male)	0.11 (-0.61, 0.83)		0.76	0.39 (-0.24, 1.01)		0.22
Relationship status (vs. single)			0.14			0.83

© 2025 Dyrbye LN et al. *JAMA Network Open*.

Married	0.47 (-0.81, 1.74)	0.47		-0.06 (-1.15, 1.03)	0.91	
Partnered	1.37 (-0.09, 2.84)	0.07		0.23 (-1.05, 1.50)	0.73	
White or Caucasian (vs. all others)	-0.45 (-1.35, 0.45)		0.32	-0.43 (-1.22, 0.36)		0.28
Not Hispanic or Latino (vs. Hispanic or Latino)	0.23 (-0.94, 1.40)		0.70	0.06 (-0.96, 1.08)		0.90
Stress (for each 1 point higher)						
Baseline stress	0.70 (0.57, 0.84)		<.001	0.76 (0.64, 0.88)		<.001
Intervention (vs. control)	-1.36 (-2.81, 0.08)		0.06	-0.29 (-1.58, 1.01)		0.66
Attending (vs. resident/fellow)	0.82 (-1.25, 2.88)		0.44	-0.15 (-2.01, 1.71)		0.87
Colorado (vs. Minnesota)	0.40 (-1.13, 1.92)		0.61	-0.37 (-1.73, 0.99)		0.59
Specialty (vs. Primary Care)			0.52			0.56
Non-Primary Care	-0.88 (-2.66, 0.89)	0.33		-0.87 (-2.46, 0.73)	0.28	
Surgical Field	-0.06 (-2.27, 2.14)	0.96		-0.604 (-2.57, 1.36)	0.54	
Work hours per week (for each additional hour)	0.01 (-0.05, 0.08)		0.69	0.01 (-0.04, 0.07)		0.66
Age in years (for each additional year)	-0.006 (-0.11, 0.10)		0.90	-0.04 (-0.14, 0.05)		0.37
Female (vs. male)	-0.27 (-1.75, 1.20)		0.72	0.50 (-0.82, 1.83)		0.46
Relationship status (vs. single)			0.31			0.03
Married	-0.41 (-3.05, 2.22)	0.76		1.20 (-1.13, 3.53)	0.31	
Partnered	1.33 (-1.71, 4.36)	0.39		3.33 (0.64, 6.03)	0.02	
White or Caucasian (vs. all others)	-0.80 (-2.66, 1.05)		0.39	-0.76 (-2.44, 0.92)		0.37
Not Hispanic or Latino (vs. Hispanic or Latino)	-0.19 (-2.62, 2.25)		0.88	-1.79 (-3.98, 0.40)		0.11
Sleepiness (for each 1 point higher)						
Baseline sleepiness	0.77 (0.67, 0.87)		<.001	0.80 (0.71, 0.90)		<.001
Intervention (vs. control)	-0.20 (-1.01, 0.62)		0.64	0.16 (-0.63, 0.96)		0.69
Attending (vs. resident/fellow)	-0.05 (-1.21, 1.12)		0.94	-0.47 (-1.61, 0.68)		0.42
Colorado (vs. Minnesota)	-0.82 (-1.66, 0.02)		0.06	-0.26 (-1.08, 0.56)		0.53
Specialty (vs. Primary Care)			0.27			0.36
Non-Primary Care	-0.72 (-1.73, 0.29)	0.16		-0.67 (-1.66, 0.31)	0.18	
Surgical Field	-0.07 (-1.33, 1.18)	0.91		-0.72 (-1.94, 0.49)	0.24	
Work hours per week (for each additional hour)	0.009 (-0.03, 0.04)		0.64	0.007 (-0.03, 0.04)		0.69
Age in years (for each additional year)	-0.01 (-0.07, 0.05)		0.70	0.002 (-0.06, 0.06)		0.95
Female (vs. male)	0.31 (-0.53, 1.14)		0.47	-0.31 (-1.13, 0.50)		0.45
Relationship status (vs. single)			0.92			0.52
Married	-0.26 (-1.76, 1.23)	0.73		-0.78 (-2.23, 0.66)	0.29	

© 2025 Dyrbye LN et al. *JAMA Network Open*.

Partnered	-0.35 (-2.07, 1.37)	0.69		-0.39 (-2.06, 1.27)	0.64	
White or Caucasian (vs. all others)	0.50 (-0.56, 1.55)		0.35	1.29 (0.26, 2.33)		0.02
Not Hispanic or Latino (vs. Hispanic or Latino)	0.05 (-1.32, 1.43)		0.94	0.23 (-1.12, 1.57)		0.74

[1] PE = parameter estimate for relevant score

© 2025 Dyrbye LN et al. *JAMA Network Open*.

eTable 4. Multivariable Models for Burnout and Resilience at 9 and 12 Months

	9 months			12 months		
	OR (95% CI)	Class p-value	Overall p-value	OR (95% CI)	Class p-value	Overall p-value
Burnout						
Baseline burnout	16.00 (6.66, 38.48)		<.001	18.37 (7.04, 47.92)		<.001
Intervention (vs. control)	1.50 (0.66, 3.44)		0.33	0.78 (0.33, 1.81)		0.56
Attending (vs. resident/fellow)	1.18 (0.33, 4.16)		0.80	2.71 (0.71, 10.38)		0.15
Colorado (vs. Minnesota)	1.16 (0.49, 2.80)		0.73	0.40 (0.16, 1.00)		0.05
Specialty (vs. Primary Care)			0.08			0.40
Non-Primary Care	0.51 (0.19, 1.34)	0.17		1.85 (0.66, 5.19)	0.24	
Surgical Field	1.70 (0.49, 5.92)	0.40		2.21 (0.61, 7.96)	0.23	
Work hours per week (for each additional hour)	1.02 (0.98, 1.05)		0.39	1.00 (0.97, 1.04)		0.86
Age in years (for each additional year)	1.01 (0.94, 1.07)		0.85	0.93 (0.86, 1.01)		0.07
Female (vs. male)	1.21 (0.53, 2.77)		0.65	0.96 (0.42, 2.23)		0.93
Relationship status (vs. single)			0.42			0.05
Married	0.90 (0.22, 3.66)	0.89		0.76 (0.18, 3.26)	0.71	
Partnered	2.15 (0.41, 11.39)	0.37		3.25 (0.67, 15.75)	0.14	
White or Caucasian (vs. all others)	1.60 (0.55, 4.65)		0.39	0.48 (0.17, 1.31)		0.15
Not Hispanic or Latino (vs. Hispanic or Latino)	1.52 (0.38, 6.08)		0.56	0.60 (0.16, 2.27)		0.45
	9 months			12 months		
	PE[1] (95% CI)	Class p-value	Overall p-value	PE (95% CI)	Class p-value	Overall p-value
Resilience						
Baseline resilience	0.72 (0.59, 0.84)		<.001	0.78 (0.68, 0.92)		<.001
Intervention (vs. control)	0.121(-1.15, 1.40)		0.85	1.07 (-0.16, 2.30)		0.09
Attending (vs. resident/fellow)	-1.54 (-3.38, 0.29)		0.10	-0.63 (-2.39, 1.12)		0.48
Colorado (vs. Minnesota)	-0.11 (-1.42, 1.21)		0.87	-1.16 (-2.42, 0.11)		0.07
Specialty (vs. Primary Care)			0.15			0.83
Non-Primary Care	0.45 (-1.10, 1.99)	0.57		-0.46 (-1.98, 1.07)	0.56	
Surgical Field	1.88 (-0.10, 3.85)	0.06		-0.47 (-2.39, 1.45)	0.63	
Work hours per week (for each additional hour)	-0.06 (-0.11, 0.00)		0.05	-0.03 (-0.08, 0.03)		0.36

© 2025 Dyrbye LN et al. *JAMA Network Open*.

Age in years (for each additional year)	0.04 (-0.05, 0.14)		0.35	-0.00 (-0.09, 0.09)		1.00
Female (vs. male)	-0.27 (-1.56, 1.02)		0.68	-0.42 (-1.67, 0.82)		0.50
Relationship status (vs. single)			0.10			0.09
Married	2.48 (0.23, 4.74)	0.03		2.38 (0.23, 4.52)	0.03	
Partnered	2.09 (-0.48, 4.67)	0.11		2.29 (-0.19, 4.76)	0.07	
White or Caucasian (vs. all others)	1.17 (-0.55, 2.89)		0.18	0.25 (-1.39, 1.89)		0.76
Not Hispanic or Latino (vs. Hispanic or Latino)	0.34 (-1.87, 2.56)		0.76	1.48 (-0.57, 3.54)		0.15

[1] PE = parameter estimate for resilience score

© 2025 Dyrbye LN et al. *JAMA Network Open*.

eTable 5. Paired Analysis: Delayed Intervention Cohort, 6 to 12 months

	6 months N = 91	12 months N = 91	P - value
Burnout			
Emotional Exhaustion, Mean (SD)[1]	22.1 (10.89)	19.3 (11.16)	<0.001
High, n (%)			0.11
Yes	32 (35.2%)	26 (28.6%)	
No	59 (64.8%)	65 (71.4%)	
Depersonalization, Mean (SD)[2]	8.0 (6.34)	6.5 (5.99)	<0.001
High, n (%)			0.001
Yes	36 (39.6%)	21 (23.1%)	
No	55 (60.4%)	70 (76.9%)	
Overall burnout, n (%) [3]			<0.001
Yes	46 (50.5%)	29 (31.9%)	
No	45 (49.5%)	62 (68.1%)	
Resilience, Mean (SD) [4]	29.5 (6.21)	30.5 (6.48)	0.04
Quality of life, Mean (SD) [5]	6.9 (1.69)	7.4 (1.51)	0.01
Depressive Symptoms, Mean (SD) [6]	5.7 (2.52)	5.7 (2.48)	0.71
Stress, Mean (SD)[7]	15.5 (6.03)	15.2 (6.70)	0.63
Sleepiness, Mean (SD)[8]	5.7 (4.20)	5.5 (4.12)	0.50

[1] The score range is 0-54; higher score indicates greater burnout symptoms. High emotional exhaustion defined by score of ≥27 on the emotional exhaustion subscale.

[2] The score range is 0-30; higher score indicates greater burnout symptoms. High depersonalization defined by score of ≥10 on the depersonalization subscale.

[3] Positive for symptoms of overall burnout if had a high score (≥27) on the emotional exhaustion and or high score (≥10) on the depersonalization subscale.

[4] The score range is 0-40, with higher scores suggesting greater resilience. Resilience was measured using the 10-item Connor-Davidson Resilience Scale.

[5] The score range is 0-10, with higher scores indicating better quality of life.

[6] The score range is 4-20, with higher scores suggesting greater depressive symptoms. Depressive symptoms was measured using the PROMIS 4a shortform.

[7] The score range is 0-40, with higher scores suggesting more stress. Stress was measured using the 10 item Perceived Stress Scale.

[8] The score range is 0-24, with higher scores suggesting more sleepiness. Sleepiness was measured using the Epworth Sleepiness Scale.

© 2025 Dyrbye LN et al. *JAMA Network Open*.

eFigure. Average Wear Time of Study Participants

(A) Average wear-time of study participants by month and study arm. Wear-time is defined as the percentage of time the participant wore the watch on their wrist over a 24-hour-period. Average wear-time was aggregated by month. (B) Average wear-time of study participants by month, study arm, and resident vs. attending status.

(A)

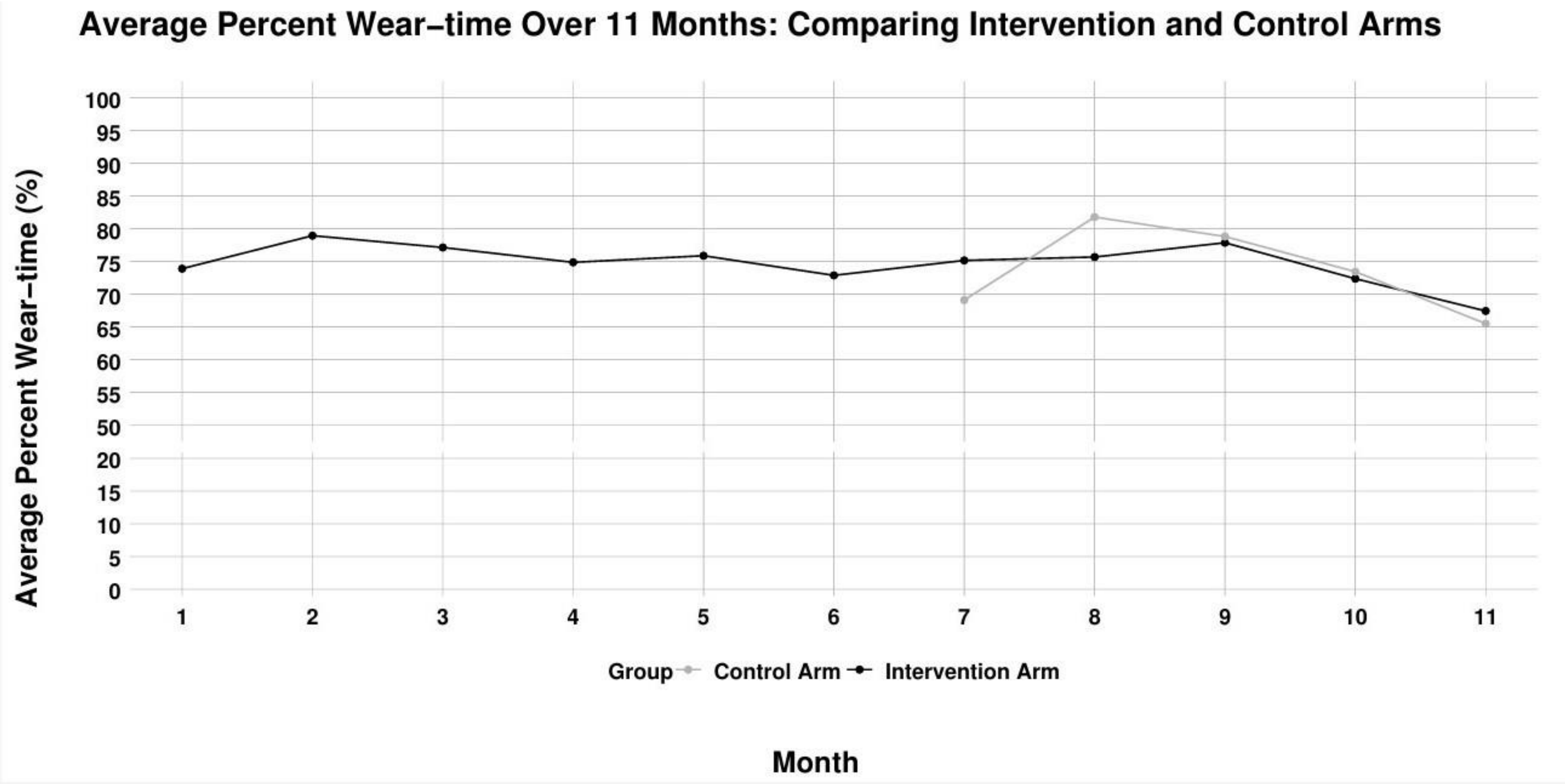

© 2025 Dyrbye LN et al. *JAMA Network Open*.

(B)

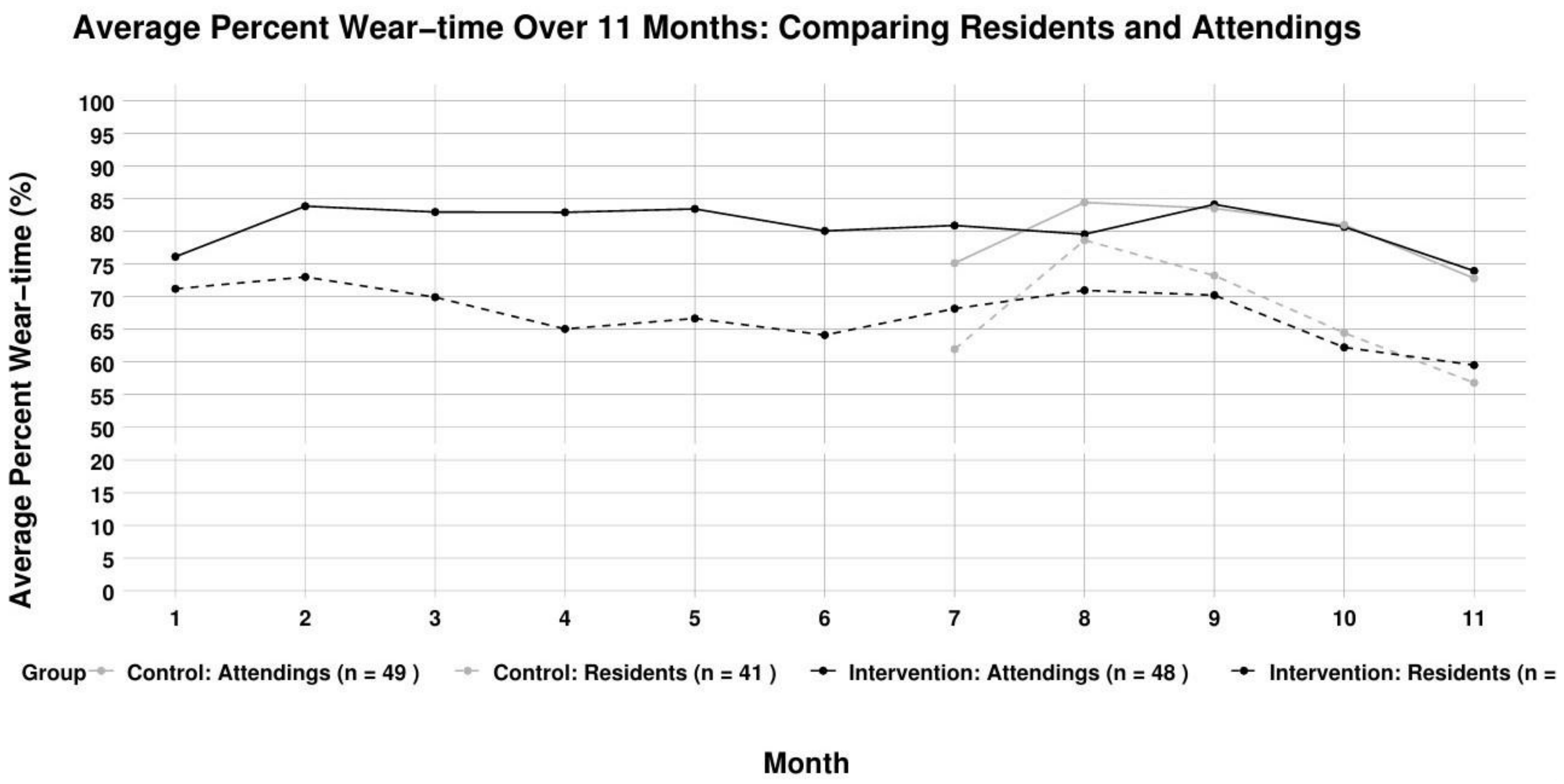

© 2025 Dyrbye LN et al. *JAMA Network Open*.
