# Supplementary material for: Smartwatch Use and Physician Well-Being: A Randomized Clinical Trial
Source: JAMA Netw Open. 2025 Aug 18;8(8):e2527275. doi: 10.1001/jamanetworkopen.2025.27275 (PMC12362228; doi:10.1001/jamanetworkopen.2025.27275)
Supplement: Supplement 3. — Data Sharing Statement [file jamanetwopen-e2527275-s003.pdf]

## Data Sharing Statement

Dyrbye. Smartwatch Use and Physician Well-Being. *JAMA Netw Open*. Published August 18, 2025. doi:10.1001/jamanetworkopen.2025.27275

**Data**
**Additional Information:** NCT05463250
**Data available:** No
